# Genome-wide assessment of DNA methylation in mouse oocytes reveals effects associated with in vitro growth, superovulation, and sexual maturity

**DOI:** 10.1186/s13148-019-0794-y

**Published:** 2019-12-19

**Authors:** Maria Desemparats Saenz-de-Juano, Elena Ivanova, Katy Billooye, Anamaria-Cristina Herta, Johan Smitz, Gavin Kelsey, Ellen Anckaert

**Affiliations:** 1 Follicle Biology Laboratory (FOBI), UZ Brussel, Vrije Universiteit Brussel, Laarbeeklaan, Brussels, Belgium; 2 Present Address: Animal Physiology, Institute of Agricultural Sciences, ETH Zurich, Zurich, Switzerland; 3 Epigenetics Programme, Babraham Institute, Cambridge, CB22 3AT UK; 4 Centre for Trophoblast Research, University of Cambridge, Cambridge, CB2 3EG UK

**Keywords:** In vitro follicle culture, Prepubertal oocytes, Superovulation, Global DNA methylation

## Abstract

**Background:**

In vitro follicle culture (IFC), as applied in the mouse system, allows the growth and maturation of a large number of immature preantral follicles to become mature and competent oocytes. In the human oncofertility clinic, there is increasing interest in developing this technique as an alternative to ovarian cortical tissue transplantation and to preserve the fertility of prepubertal cancer patients. However, the effect of IFC and hormonal stimulation on DNA methylation in the oocyte is not fully known, and there is legitimate concern over epigenetic abnormalities that could be induced by procedures applied during assisted reproductive technology (ART).

**Results:**

In this study, we present the first genome-wide analysis of DNA methylation in MII oocytes obtained after natural ovulation, after IFC and after superovulation. We also performed a comparison between prepubertal and adult hormonally stimulated oocytes. Globally, the distinctive methylation landscape of oocytes, comprising alternating hyper- and hypomethylated domains, is preserved irrespective of the procedure. The conservation of methylation extends to the germline differential methylated regions (DMRs) of imprinted genes, necessary for their monoallelic expression in the embryo. However, we do detect specific, consistent, and coherent differences in DNA methylation in IFC oocytes, and between oocytes obtained after superovulation from prepubertal compared with sexually mature females. Several methylation differences span entire transcription units. Among these, we found alterations in *Tcf4*, *Sox5*, *Zfp521*, and other genes related to nervous system development.

**Conclusions:**

Our observations show that IFC is associated with altered methylation at specific set of loci. DNA methylation of superovulated prepubertal oocytes differs from that of superovulated adult oocytes, whereas oocytes from superovulated adult females differ very little from naturally ovulated oocytes. Importantly, we show that regions other than imprinted gDMRs are susceptible to methylation changes associated with superovulation, IFC, and/or sexual immaturity in mouse oocytes. Our results provide an important reference for the use of in vitro growth and maturation of oocytes, particularly from prepubertal females, in assisted reproductive treatments or fertility preservation.

## Background

Recent progress in stem cell biology has opened up the possibility of generating mature gametes in vitro from pluripotent cells, with the demonstration that the entire cycle of the female mouse germline can be reproduced in vitro [1]. It is essential to prove the safety of such procedures before they become applied in human [2]. Up to now, only in vitro maturation (IVM) of oocytes from small antral follicles (2–8 mm) has seen a successful clinical application [3], but efforts are being made to design new culture systems capable of supporting the in vitro growth of early-stage follicles toward competent oocytes [4–6]. For example, multi-step culture models have been developed to support the ex vivo propagation of human immature oocytes from primordial/unilaminar stages to the metaphase-II (MII) stage [7].

Although most babies conceived by assisted reproductive technologies (ARTs) seem healthy, studies in various species have reported phenotypic or functional alterations associated with ART procedures [8]. It has also been shown in animal models that a suboptimal environment around the time of conception can predispose offspring to adverse metabolic and cardiovascular phenotypes [9–11]. Furthermore, a number of studies have reported an increased risk of genomic imprinting disorders in ART children, including Beckwith-Wiedemann (BWS), Angelman (AS), Prader-Willi (PWS), and Silver-Russell (SRS) syndromes [12–17], although the extent to which ART procedures themselves or the underlying fertility impairments of parents contribute is not fully resolved [16, 18–20]. DNA methylation alterations have been identified as possible underlying mechanisms, but there is no definitive knowledge about the impact of ARTs on DNA methylation establishment in oocytes.

In mice, de novo DNA methylation in oocytes starts around 10 days after birth and is almost complete by the fully-grown germinal vesicle (GV) stage [21, 22]. Methylation acquisition depends on the de novo DNA methyltransferases DNMT3A and DNMT3L [22] and occurs progressively from the secondary follicle stage as the oocyte increases in diameter [23]. The resulting oocyte methylome is unique and highly structured, divided into highly methylated domains and unmethylated domains, with methylation predominantly intragenic and associated with transcriptionally active gene bodies [24]. Included in this gene-body methylation are the CpG islands (CGIs) that constitute the germline differential methylated regions (gDMRs) of imprinted genes necessary for their parent-of-origin monoallelic expression after fertilization [24, 25]. The link between oocyte transcription events and de novo methylation suggests the possibility that transcriptional abnormalities could result in DNA methylation errors.

Studies that have surveyed a limited number of imprinted genes suggest that in vitro follicle culture (IFC) and superovulation do not impair the establishment of methylation at imprinted genes [26, 27]. We described normal methylation patterns for the gDMRs of *H19*, *Snrpn*, *Igf2r*, and *Mest* in mouse metaphase-II (MII) oocytes obtained after culture from the early preantral follicle stage under various culture conditions and treatments [26–28]. Similar results have been observed for the *H19*/*IGF2*, *PEG3*, and *SNRPN* gDMRs in bovine IVM [29] and the *LIT1*, *SNRPN*, *PEG3*, and *GTL2* gDMRs in human IVM [30]. However, genome-wide analysis has revealed that apart from the classical imprinted gDMRs, a large number of other CGIs become highly methylated in oocytes [22, 25], some of which may be important for gene regulation in the embryo. Notably, oocyte-derived methylation outside of imprinted genes plays a major regulatory role in the trophoblast lineage in mouse [31], and determines placental-specific imprinting in human [32, 33]. Despite the fact that DNA methylation establishment at imprinted gDMRs in the oocyte proceeds normally, there is accumulating evidence that superovulation and IFC alters maintenance of gDMR methylation during embryo development [34–37]. A possible explanation is that culture and superovulation affect maternal-effect factors required for imprinting maintenance after fertilization.

Here, we generated high-resolution, genome-wide methylation maps of oocytes derived from follicle culture and oocytes obtained after superovulation. Our results indicate that the oocyte methylome is robust and not grossly altered by these ART-related procedures. However, we do find significant gene-specific differences associated in particular with IFC and with sexual maturity. Our data provide an essential reference for epigenetic safety assessments in studies that aim to improve and optimize oocyte culture systems. In addition, they provide new insights into oocyte methylation at prepubertal stages that could be important for improvement of fertility preservation programs.

## Results

### Experimental design and properties of in vitro and in vivo derived oocytes

**Fig. 1:**
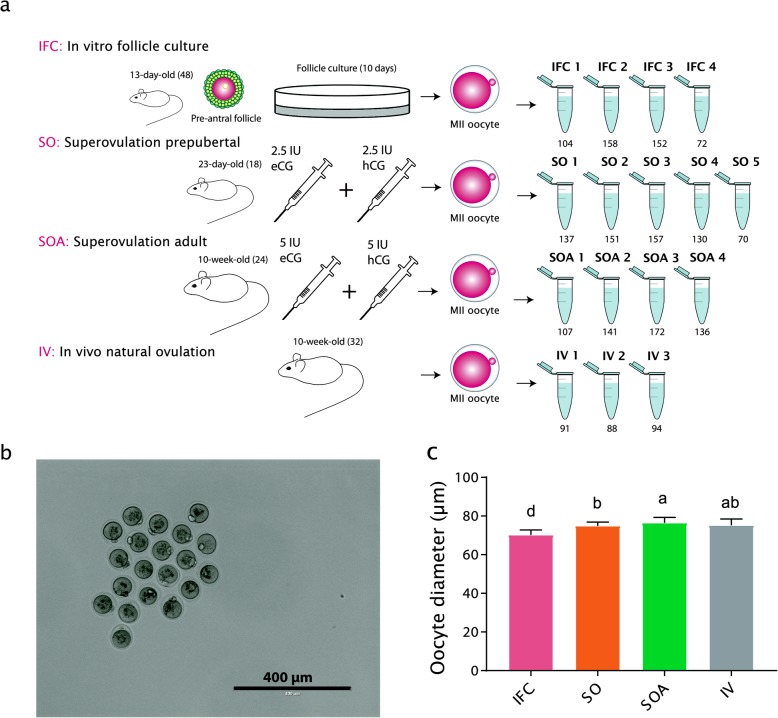
**a** Experimental groups used for the genome-wide DNA methylation analysis. The number of females used per group is indicated in brackets next to their age. The tubes represent the number of biological replicates and the number under each tube indicates the number of oocytes pooled in each sample. *MII* metaphase II, *eCG* equine chorionic gonadotropin, *hCG* human chorionic gonadotropin. **b** MII oocytes from IFC obtained after 10 days of culture. Pictures were taken before snap freezing to measure oocyte diameters. **c** Oocyte diameter per group. Bar charts show the mean and the standard deviation (SD). Lower-case letters denote significant differences (*p* < 0.05) after applying non-parametric Krustall-Wallis and Dunn’s multiple comparisons tests

The current study aimed to evaluate the effects of procedures associated with ARTs on DNA methylation establishment in mouse oocytes by performing genome-wide bisulphite sequencing of MII oocytes obtained after preantral follicle culture (IFC) and superovulation compared with natural ovulation (Fig. 1a). MII oocytes were selected in order to analyze those oocytes that had successfully completed nuclear maturation after preantral and antral development in vitro. Because sexual maturity of the mouse strain used in this study is only attained after 4 weeks [38], age-matched oocytes were used for the assessment of the effect of follicle culture and superovulation. Therefore, our study design comprised four groups. Preantral follicles from prepubertal 13-day-old female mice were cultured for 10 days in an established follicle culture system [39] to obtain the IFC group, which were compared with prepubertal superovulated 23-day-old females (SO). In addition, superovulated oocytes from adult females (SOA; 10 weeks old) were compared to adult naturally ovulated oocytes (in vivo, IV; 10 weeks old). Therefore, the comparisons also enable the effects of sexual maturity and hormonal stimulation on the oocyte methylome to be evaluated. In all cases, oocytes were from F_1_ (C57BL/6JxCBA/Ca) females, so they were genetically identical.

IFC MII oocytes were obtained from six independent culture experiments of 10 days duration. In each culture, 200 preantral follicles were cultured, of which 31.1 ± 2.53% (mean ± SEM) attained the antral stage. The polar body extrusion (PB) rate after r-hCG/r-EGF stimulation of antral follicles was 83.94 ± 2.36%. For the MII oocytes obtained from female mice, the average number of oocytes retrieved per female after superovulation was higher in prepubertal (37.7 ± 5.95; mean ± SEM) than in adult females (23.0 ± 0.67, mean ± SEM). This can be explained by the fact that at day 23 ovaries contain more synchronized follicles from the first wave in the early antral stage that are responsive to stimulation. For IV MIIs collected after natural ovulation, 6–9 oocytes were isolated per female.

DNA methylation acquisition in the oocyte correlates with increasing diameter [23]. Therefore, before snap freezing, pools of oocytes were photographed in order to evaluate their sizes (Fig. 1b). IFC oocytes had a significantly smaller diameter than the other groups (70.17 ± 0.11 μm; mean ± SEM, Fig. 1c), SOA oocytes had significantly greater diameters than their prepubertal (SO) counterparts (74.85 ± 0.05 μm vs. 76.42 ± 0.10 μm; mean ± SEM), but IV oocytes (75.2 ± 0.4 μm; mean ± SEM) were not significantly different from SO or SOA oocytes. In the maternal strain, C57BL/6 J de novo methylation is expected to be substantially completed once oocytes attain a diameter of ≥ 70 μm [23].

### A conserved pattern of genomic DNA methylation in oocytes irrespective of in vitro or in vivo protocol

For genome-wide DNA methylation analysis, MII oocytes retaining their polar bodies were collected in pools of between 70 and 172 oocytes, and between three and five pools per condition (Fig. 1a). Whole-genome DNA methylation maps were generated using the post-bisulphite adapter tagging (PBAT) method with previously described modifications [40, 41]. After alignment and removal of sequence read, duplicates between 6,357,771 and 29,532,884 uniquely mapped reads were obtained per library (Additional file 8: Table S1). When the replicates were merged within the four experimental groups, between 36,244,782 and 75,743,443 reads were obtained per group, resulting in a coverage of CpGs (≥ 1 read) in the merged groups of between 62.95% and 77.4% (Additional file 9: Table S2).

**Fig. 2:**
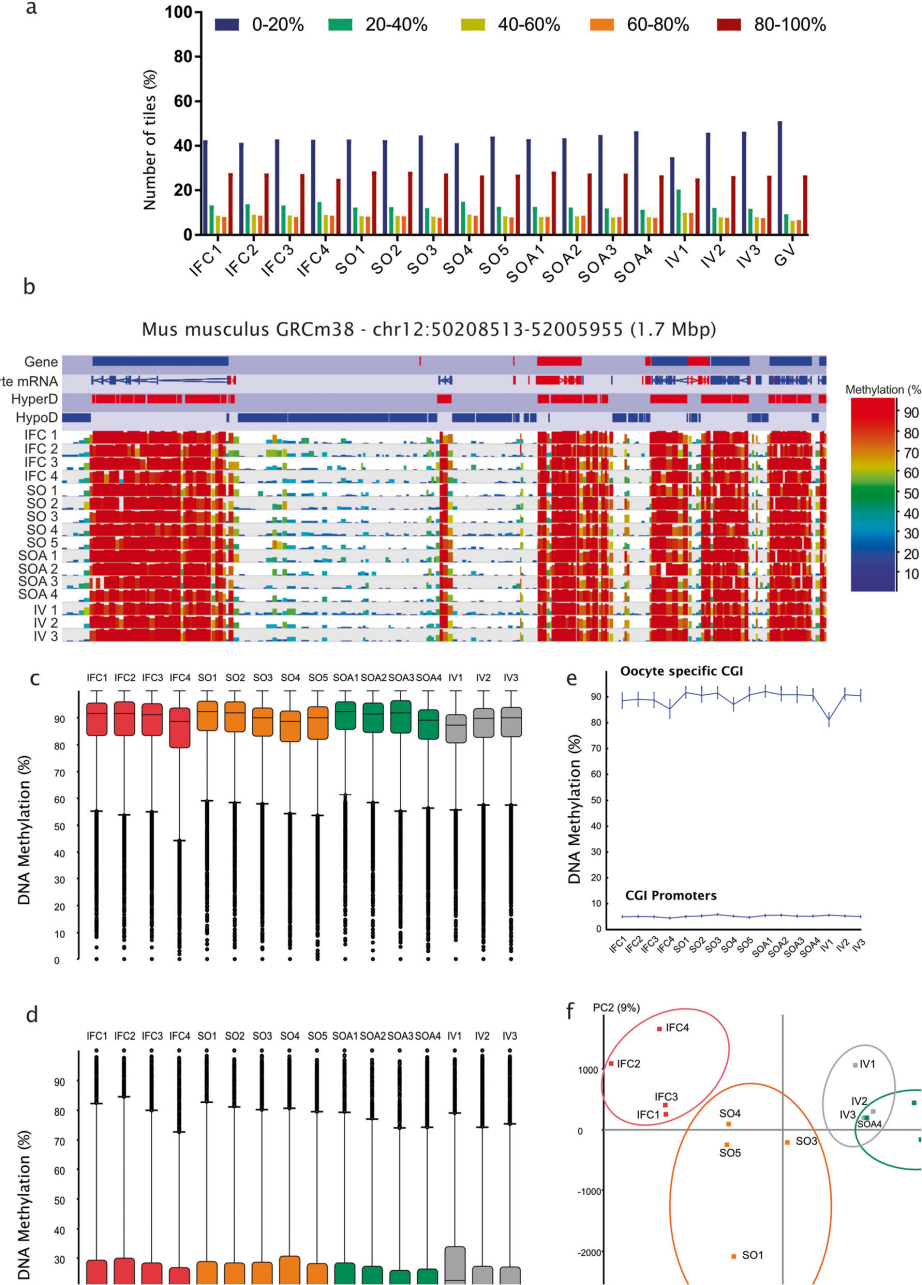
**a** Distribution of DNA methylation across the genome in 100-CpG windows in all samples compared to Germinal Vesicle (GV) oocytes from Shirane et al. [38]. **b** SeqMonk screenshot of a 1.7 Mb region of chromosome 11 depicting the hypermethylated (HyperD) and hypomethylated (HypoD) domains characteristic of the oocyte methylome in each of the 16 individual methylation datasets. Genes and oocyte mRNA are shown in red or blue depending on their direction of transcription (forward and reverse, respectively). Each color-coded vertical bar in the screenshot represents the methylation value of a non-overlapping 100 CpG tile. HypoD, HyperD, and oocyte mRNA annotation tracks are derived from Veselovska et al. [24]. **c** DNA methylation percentages at HyperD in all samples (*n* = 26,570). In the box:whiskers plot, the line across the middle of the box shows the median, the upper and lower extremities of the box show the 25th and 75th percentile of the set of data, and the upper and lower black whiskers show the median plus/minus the interquartile (25–75%) range multiplied by 2. Individual points which fall outside this range are shown as filled circles, and represent single outlier tiles. **d** Box:whisker plot showing the DNA methylation percentages at HypoD (*n* = 38,739). **e** DNA methylation percentages of CpG Islands (CGI) located at promoters (*n* = 11,542) and CGIs highly methylated in oocytes (*n* = 2014). Each point represents the mean value along with error bars indicating the 95% confidence interval for the measure. **f** Principal component analysis (PCA) of informative 100-CpG tiles (value between 0 and 100 in all 16 samples; *n* = 195,170) shows how biological replicates cluster together within each group and differently between conditions

Total genomic CpG methylation increases from 2.3% in non-growing oocytes to 38.7% at the fully grown GV stage; in addition, oocytes accumulate higher levels of methylation of non-CpG cytosines than most somatic tissues [42]. Global CpG methylation in all our samples was 37.7–42.9% (Additional file 8: Table S1), and non-CpG methylation (CHG and CHH) was 3.2–5.2%, in line with expectations (Additional file 8: Table S1). To evaluate the genomic methylation profile in detail, we generated fixed size tiles of 100 CpGs that segregated the genome into 218,689 non-overlapping tiles. From these we obtained 195,710 tiles with coverage in all 16 samples. We first observed that all the replicates were highly correlated (Additional file 1: Figure S1). The distribution of methylation levels of these tiles across the genome is highly bimodal, similar to previously published data [22, 24, 42, 43], with most 100 CpG tiles having less than 20% (43.5 ± 0.79%; mean ± SEM) or greater than 80% (27.1 ± 0.23%; mean ± SEM) methylation (Fig. 2a). These results confirm the absence of contamination by cumulus cells, although one possible exception was the naturally ovulated sample IV1, which had a lower percentage of hypomethylated (0–20%) tiles and higher percentage of intermediately methylated tiles (20–40%) (Fig. 2a). Browser screenshots for this sample also indicated a marginally higher level of methylation in regions ordinarily unmethylated in oocytes (Fig. 2b). However, we considered this acceptable, particularly given the difficulty in obtaining significant numbers of naturally ovulated oocytes, and including the sample would benefit statistical analysis of the datasets. In addition, there was no evidence that this sample impaired the subsequent identification of group-specific methylation differences (below).

A specific feature of the oocyte methylome is its division into hypermethylated (HyperD) and hypomethylated (HypoD) domains that are respectively correlated with active transcription units, and intergenic or inactive genomic regions [24]. As Fig. 2c shows, we also observed this pattern of methylation in all samples. The mean methylation levels of HyperDs in the informative tiles from all samples ranged from 84.7 to 89.4% (Fig. 2c), while for HypoDs it was 16.1 to 24.3% (Fig. 2d). Methylation of CGIs in oocytes is also of significance: although most are hypomethylated, a defined subset of ~ 2000 CGIs gain high levels of methylation, including the gDMRs of imprinted loci [22, 25]. Among 23,018 CGIs in the mouse genome (Illingworth et al. 2010), promoter-associated CGIs (11542) were similarly hypomethylated in all samples, whilst oocyte-specific methylated CGIs (2014) exhibited high levels of methylation, as expected (Fig. 2e). We also evaluated methylation levels at other genomic features, such as gene bodies, intergenic regions, and promoters, which also revealed highly similar mean methylation levels in all samples (Additional file 2: Figure S2). We also looked at various classes of mouse repetitive elements (LINE, SINE, LTR, and satellite repeats). The low coverage of these regions by uniquely mapped reads did not allow us to assess methylation in individual samples, but merging the data per group showed that these elements also had similar global methylation between groups (Additional file 2: Figure S2).

The results above revealed that the DNA methylation landscape and methylation over most genome annotations were globally similar in the four experimental groups. However, principal component analysis (PCA) indicated that there was variation between groups that allowed them to be clustered, particularly along PC1 (Fig. 2f); specifically, the IV and SOA groups clustered together, while the SO and IFC samples clustered with their own biological replicates but separately from each other. Note that sample IV1 clustered with the other two naturally ovulated samples (IV2, IV3), validating our decision to retain this sample in our analysis. The results of the PCA suggest that there are specific and consistent methylation differences between experimental groups.

### In vitro growth from the preantral stage results in hypomethylation of a discrete set of loci in MII oocytes

**Fig. 3:**
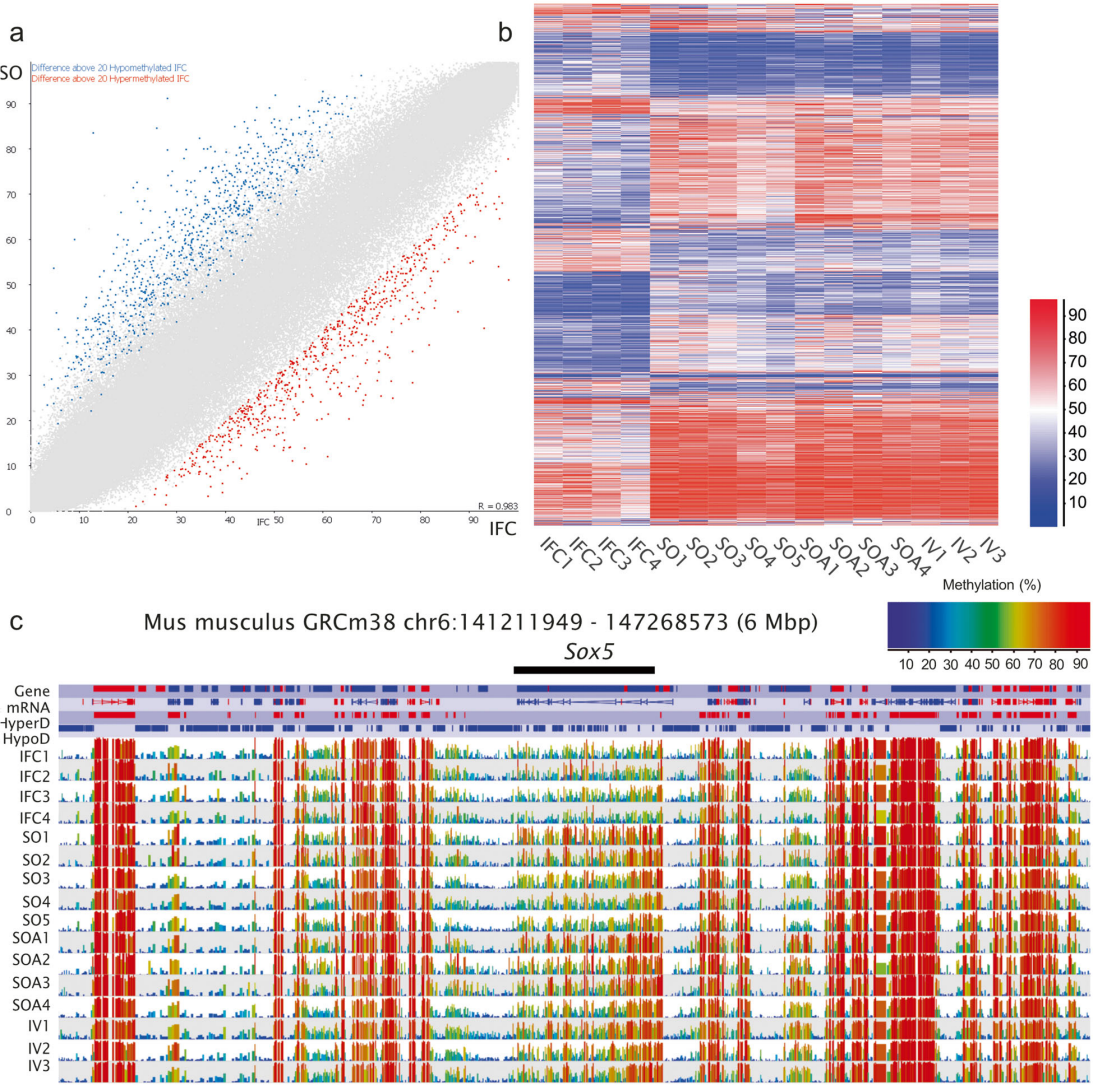
**a** Scatterplot for informative tiles (100 CpG window size, *n* = 195,170) in both IFC and SO. Data from replicates are pooled. Differentially methylated tiles (*p* < 0.05) identified by logistic regression and with a methylation difference of ≥ 20% are highlighted in blue or red (hypomethylated in IFC and hypermethylated in IFC, respectively). **b** Heat map after unsupervised hierarchical clustering of all differentially methylated tiles (*p* < 0.05, 100-CpG window size, *n* = 6362) between IFC and SO. The heatmap shows how biological replicates were consistent within groups and IFC differed in a similar way from SO, SOA, and IV for these differentially methylated sites. **c** SeqMonk screenshot of a 6 Mbp region of chromosome 6 showing methylation at the *Sox5* locus, with 18 hypomethylated tiles in IFC. Each color-coded vertical bar in the screenshot represents the methylation value of a non-overlapping 100-CpG tile. Genes and oocyte mRNA are shown in red or blue depending on their direction of transcription (forward or reverse, respectively)

We sought first to identify differential methylated regions (DMRs) between in vitro and correspondingly aged in vivo developed and superovulated oocytes (IFC vs. SO). Using logistic regression analysis, we identified 6362 significantly different 100-CpG tiles from 199,138 informative tiles (Table 1, 3.2% of the total; *p* < 0.05 after Benjamini-Hochberg correction for multiple testing). Of these significant tiles, 1531 (24.1%) had greater than 20% methylation difference: 829 hypomethylated and 702 hypermethylated in IFC (Fig. 3, Additional file 10: Table S3). Hierarchical cluster analysis of the samples based on this set of DMRs separated the IFC samples from the SO, SOA, and IV samples (Fig. 3b), suggesting an effect specifically of the follicle culture system. Of the tiles hypomethylated by at least 20%, there was a deficiency of tiles overlapping promoters, while hypermethylated tiles were enriched in gene bodies and promoters (Additional file 3: Figure S3).

**Table 1:** Differentially methylated tiles found in all pair-wise comparisons

Comparison	Informative tiles	Differentially methylated tiles	20% difference hypomet	20% difference hypermeth
IFC vs. *SO*	199,138	6362 (3.2%)	829	702
SO vs. *SOA*	197,317	14,795 (7.5%)	48	2031
SOA vs. *IV*	199,821	1248 (0.6%)	455	110
IFC vs. *SOA*	197,565	17,982 (9.1%)	4158	3477

The experimental group in italic is considered the control in the comparison, while the other groups is hyper or hypomethylated

Although most DMRs were dispersed across the genome (73.1%), some specific loci contained multiple differential methylated tiles (Additional file 11: Table S4). There were 17 genes with at least 5 hypomethylated tiles in IFC, those with the greatest number of hypomethylated tiles included the SRY-box containing gene 5 (*Sox5*, 18 tiles, Fig. 3c), Enhancer trap locus 4 (*Etl4*, 14 tiles, Additional file 4: Figure S4A), and myosin XVI (*Myo16*, 13 tiles, Additional file 4: Figure S4B). The SOX protein family represents important developmental regulators; in particular, transcription factor SOX5 regulates the activity of *Sox9* and *Sox10* during development of chondrocytes, oligodendrocytes, and neurons, among other cell types [44]. *Etl4* is expressed in the notochord of early embryos and in multiple epithelia during later development [45]. Finally, myosins are a family of ATP-dependent motor proteins responsible for actin-based motility. Some myosins are proposed to have nuclear functions, including chromatin remodeling, RNA transport, facilitation of long-range chromosome movement, and RNA polymerase-mediated transcription [46]. *Myo16* expression may regulate the cell cycle, and increased *Myo16* expression is necessary for resumption of S-phase progression [46]. These concerted, gene-specific effects we observe could be consistent with methylation changes downstream of transcriptional differences between the IFC and the other oocyte groups. Gene ontology (GO) enrichment analysis of these 17 hypomethylated genes revealed that the most altered biological processes were nervous system development and neuron differentiation (Table 2).

**Table 2:** Enriched biological processes of hypomethylated genes in IFC compared to SO (> 20% methylation difference, *n* = 17)

#term ID	Term description	Observed gene count	False discovery rate
GO:0007399	Nervous system development	8	0.0256
GO:0030182	Neuron differentiation	6	0.0256
GO:0032501	Multicellular organismal process	12	0.0256
GO:0048666	Neuron development	5	0.0256
GO:0048699	Generation of neurons	7	0.0256
GO:0048731	System development	11	0.0256
GO:0007417	Central nervous system development	5	0.0272
GO:0021953	Central nervous system neuron differentiation	3	0.0272
GO:0021955	Central nervous system neuron axonogenesis	2	0.0272
GO:0048169	Regulation of long-term neuronal synaptic plasticity	2	0.0272
GO:0050850	Positive regulation of calcium-mediated signaling	2	0.0312
GO:0060291	Long-term synaptic potentiation	2	0.0355
GO:0023052	Signaling	9	0.0414
GO:0000902	Cell morphogenesis	4	0.0496
GO:0007154	Cell communication	9	0.0496
GO:0031646	Positive regulation of neurological system process	2	0.0496
GO:0048709	Oligodendrocyte differentiation	2	0.0496

In comparison, there were only four loci with multiple hypermethylated tiles in IFC. These included *Prdm16* (PR domain containing 16, Additional file 5: Figure S5A) with eight differentially methylated tiles and *Soga 1* (suppressor of glucose autophagy associated, Additional file 5: Figure S5B), and *Map2k6* (mitogen-activated protein kinase kinase 6) both with 6 differently methylated tiles (Additional file 11: Table S4). PRDM16 is a transcription factor that can interact with many different proteins and is critical for the modulation of multiple signaling pathways, including transforming growth factor beta (TGFβ) and bone morphogenic protein (BMP) [47]. It was revealed to be inappropriately expressed in (1;3)(p36;q21)-positive leukemia cells and, apart from its role in cell proliferation, it can also drive certain tissue-specific differentiation in brown adipose tissue (BAT) [47–49].

To interrogate CGIs specifically, we designed tiles around CGIs and increased the threshold for minimum observations to 20. Results showed that there were 85 CGIs with significantly different levels of methylation between IFC and SO (*p* < 0.05, Additional file 10: Table S3). From those CGIs, 15 were hypomethylated CGI with a difference ≥ 20% (Table 3): ten were overlapping promoter regions, five were inside gene bodies, and one was intergenic. Interestingly, 11 of those CGIs were not detected previously with the 100 CpG window unbiased analysis (Table 3). GO enrichment analysis found that the 15 genes associated with these CGIs are involved in KEGG Pathways in cancer (mmu05200).

**Table 3:** Differentially methylated CGI tiles found in all pair-wise comparisons

Comparison	Total CGI tiles (*p* value < 0.05)	Genes associated with Hypomethylated CGI (≥ 20% methylation difference)	Genes associated with Hypermethylated CGI (≥ 20% methylation difference)
IFC vs. **SO**	85	*Fzd5*, *Phf19*, *Kctd8*, *Repin1*, *Iqsec1*, *Foxp1*, *Fgfr1*, *Cnot7,* *Gm20388*, *Col13a1*, *Jmjd1c*, *Foxred2*, *Ccnd3*, *Kcnn2*, *Csf2ra*	*Qtrt1*, *Fam100b*, *Pick1*
SO vs. **SOA**	107	*Slc12a5, Kctd8, Fbxl3*	*Casz1*, *Ncor1*, *Tmem121*, *Ryr2*, *Sfrp5*
SOA vs. **IV**	60	–	*Nkpd1*, *Bcan*, *Krt7*, *Ncor1*, *Golim4*, *Manf*

The experimental group in bold is considered the control in the comparison, while the other group is hyper or hypomethylated. The underlined gene names are those previously identified by the 100 CpG window unbiased analysis

### Minimal effect of hormonal stimulation on DNA methylation in oocytes from adult females

Using similar approaches, we compared the age-matched SOA and IV groups and found 1248 significantly different tiles (*p* < 0.05) from 199,821 informative tiles (Table 1, 0.6%). After filtering for significant tiles with ≥ 20% difference, there were only 110 hypomethylated and 455 hypermethylated tiles in SOA (Additional file 12: Table S5). Particularly for tiles hypermethylated in SOA, there was a strong enrichment in promoters compared with the genome average (*n* = 256, Additional file 3: Figure S3). GO functional annotation analysis revealed that the genes associated with those promoters were enriched in nitrogen compound metabolic processes and DNA repair (Additional file 13: Table S6). Contrary to what we observed above, the SOA vs. IV differentially methylated tiles were dispersed throughout the genome and the only genes with more than a single differently methylated tile were sidekick homolog 1 (*Sdk1*, 2 hypomethylated tiles), latrophilin 2 (*Lphn2*, 2 hypermethylated tiles), cadherin 13 (*Cdh13*, 2 hypermethylated tiles), and transcription factor 4 (*Tcf4*, 2 hypermethylated tiles) suggesting very few regions of concerted methylation difference associated with superovulation of adult females (Additional file 13: Table S6). We could regard this comparatively low number of mainly dispersed differential methylated tiles as representing false discovery, which could give us an empirical background false discovery rate that increases confidence in the DMRs identified in the other comparisons. With the CGI-specific analysis, we identified 60 differentially methylated CGIs (Additional file 12: Table S5), but only six were hypermethylated with a ≥ 20% difference (Table 3). An intragenic CGI in the gene NTPase KAP family P-loop domain containing 1 (*Nkpd1*) was the only differentially methylated CGI not overlapping a promoter.

### Consistent DNA methylation differences in prepubertal oocytes

**Fig. 4:**
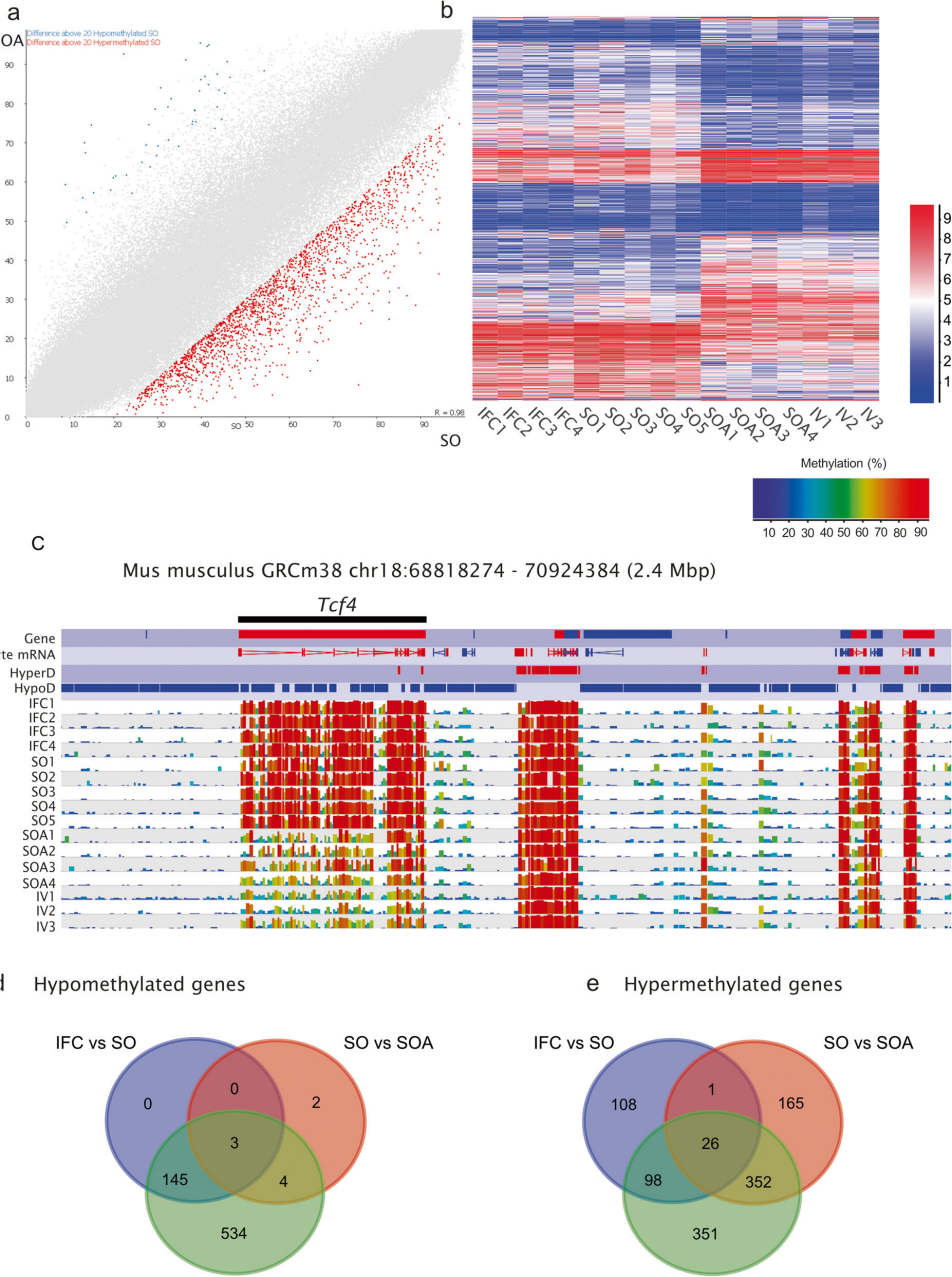
**a** Scatterplot for common informative tiles (100 CpG window size, *n* = 195,170 between SO and SOA. Data from replicates are pooled. Differentially methylated tiles (*p* < 0.05) identified by logistic regression and with a methylation difference of ≥ 20% are highlighted in blue or red (hypomethylated in IFC and hypermethylated in IFC, respectively). **b** Heat map after unsupervised hierarchical clustering of all differentially methylated tiles (*p* < 0.05, 100 CpG window size, *n* = 14,795 between SO and SOA. The heatmap shows that the IFC group followed the same trend as SO, while the IV group was similar to SOA for these differentially methylated sites. **c** SeqMonk screenshot showing the methylation levels at Tcf4 locus (with 28 hypermethylated tiles). Each color-coded vertical bar represents the methylation value of a non-overlapping 100-CpG tile. Genes and oocyte mRNA are shown in red or blue depending on their direction of transcription (forward or reverse, respectively). **d**, **e** Venn diagrams showing the common hypomethylated and hypermethylated genes that were affected in IFC vs. SO, SO vs. SOA, and IFC vs. SOA

The developmental potential of oocytes from prepubertal animals is inferior to that of oocytes from adult animals [50]. For that reason, we compared the methylome of prepubertal and adult oocytes obtained after hormonal stimulation (SO, SOA). Both groups were superovulated using the same treatment (eCG followed by hCG). In this comparison, we detected 14,795 differential methylated tiles from 197,317 informative tiles (Table 1, 7.5%; *p* < 0.05), which was the highest of any of our comparisons. Of these, there was a strong bias toward hypermethylation, with 2031 hypermethylated by ≥ 20% in SO, and only 48 hypomethylated by ≥ 20% (Fig. 4a, Additional file 14: Table S7). Hierarchical cluster analysis of these differences showed that the IFC group followed the same trend as SO, while the IV group was similar to SOA (Fig. 4b), suggesting a consistent effect of sexual maturity or age on DNA methylation. The proportion of hypermethylated tiles overlapping gene bodies was higher than expected (*p* < 0.05; Additional file 3: Figure S3).

The 48 hypomethylated tiles were mostly dispersed across the genome, although 14.6% of the hypomethylated tiles were located in the gene *Soga1* (seven tiles; Additional file 15: Table S8; Additional file 5: Figure S5). *Soga1* encodes a microtubule associated protein known to be involved in glucose and glycogen metabolism [51]. Conversely, 40 genes contained multiple hypermethylated tiles in SO (≥ 5 tiles; Additional file 15: Table S8). Interestingly, we also found that 81.2% of the clustered tiles within gene bodies were located in HypoD regions, suggesting concerted methylation gains in superovulated prepubertal oocytes that could be associated with age-related transcriptional differences. The GO enrichment analysis of the 40 hypermethylated genes revealed that the most altered pathway was single organism signaling (16 genes), followed by neuron-neuron synaptic transmission (four genes, Table 4). The most affected genes were *Tcf4* (28 tiles, Fig. 4c), zinc finger protein 521 (*Zfp521*, 26 tiles, Additional file 6: Figure S6A), and attractin like 1 (*Atrnl1*, 22 tiles, Additional file 6: Figure S6B). *Tcf4* encodes an E-box protein crucial for development of the mammalian nervous system. Haploinsufficiency of *TCF4* in humans causes the Pitt–Hopkins mental retardation syndrome, and other members of the gene family are not able to compensate for its absence during the differentiation of progenitors of the pontine neurons [52]. *Zfp521* codes for a zinc finger DNA binding protein implicated in the function and differentiation of early progenitor cells in neural and adipose tissues, the erythroid lineage, and bone development, and is involved in neuronal development and differentiation [53, 54].

**Table 4:** Enriched biological processes of hypermethylated genes in SO compared to SOA (> 20% methylation difference, *n* = 40)

#term ID	Term description	Observed gene count	False discovery rate
GO:0048846	Axon extension involved in axon guidance	3	0.0012
GO:0009987	Cellular process	35	0.0027
GO:0050919	Negative chemotaxis	3	0.0125
GO:0070100	Negative regulation of chemokine-mediated signaling pathway	2	0.0125
GO:0021825	Substrate-dependent cerebral cortex tangential migration	2	0.0135
GO:0035385	Roundabout signaling pathway	2	0.0135
GO:0060560	Developmental growth involved in morphogenesis	4	0.0135
GO:0023052	Signaling	17	0.0139
GO:0007154	Cell communication	17	0.0185
GO:0007268	Chemical synaptic transmission	5	0.0185
GO:0042118	Endothelial cell activation	2	0.0185
GO:1902668	Negative regulation of axon guidance	2	0.0185
GO:0003180	Aortic valve morphogenesis	2	0.0244
GO:0022008	Neurogenesis	10	0.0244
GO:0001657	Ureteric bud development	3	0.0322
GO:0001964	Startle response	2	0.0387
GO:0007399	Nervous system development	11	0.0387
GO:0035235	Ionotropic glutamate receptor signaling pathway	2	0.0387
GO:0048699	Generation of neurons	9	0.0387
GO:0050789	Regulation of biological process	27	0.0387
GO:0050794	Regulation of cellular process	26	0.0387
GO:0090287	Regulation of cellular response to growth factor stimulus	4	0.0387
GO:0120033	Negative regulation of plasma membrane bounded cell projection assembly	2	0.0387
GO:0008045	Motor neuron axon guidance	2	0.0398
GO:0030182	Neuron differentiation	7	0.0398
GO:0030517	Negative regulation of axon extension	2	0.0398
GO:0035249	Synaptic transmission, glutamatergic	2	0.0398
GO:0090288	Negative regulation of cellular response to growth factor stimulus	3	0.046
GO:0007267	Cell-cell signaling	6	0.049
GO:0021884	Forebrain neuron development	2	0.0494

**Fig. 5:**
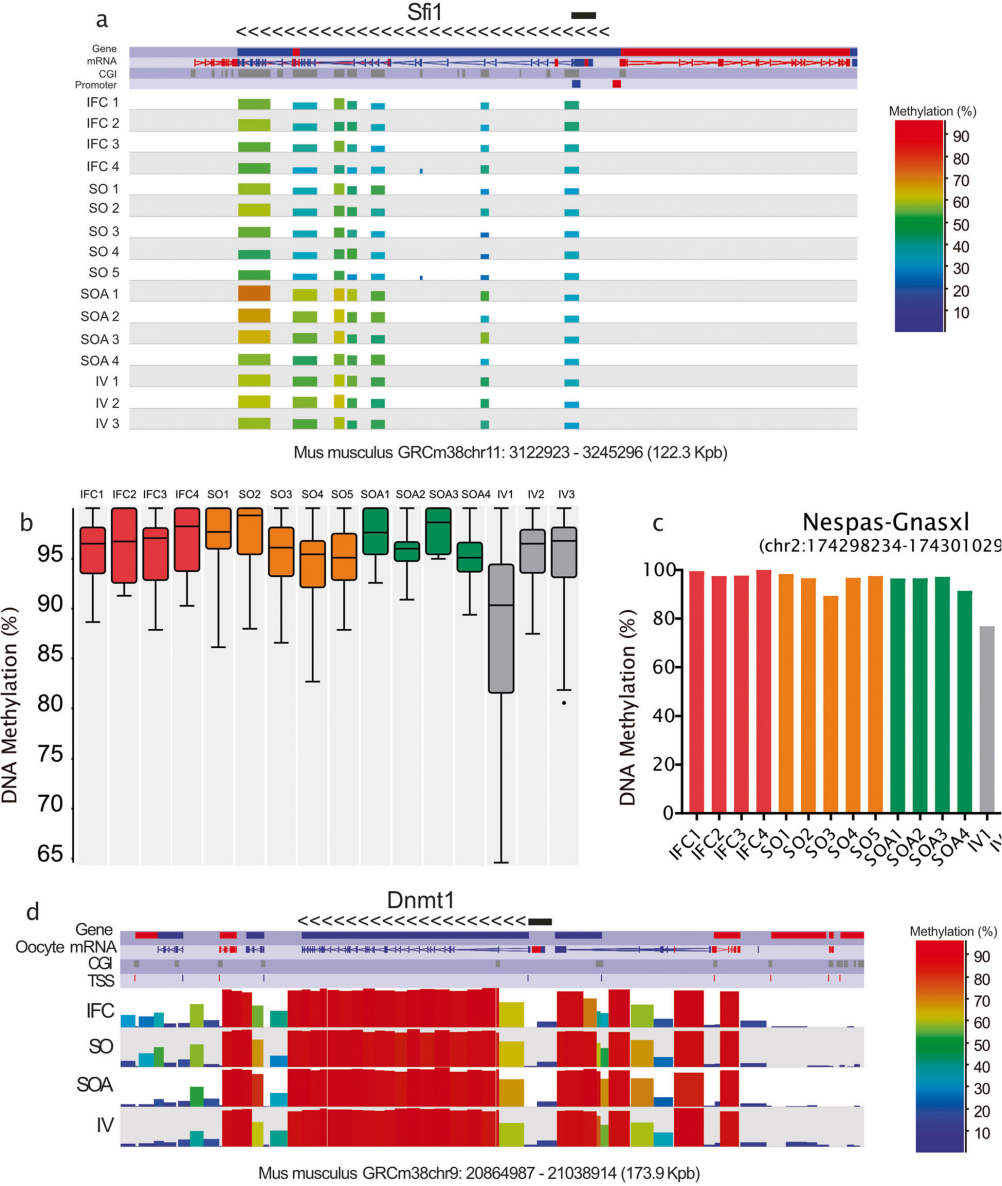
**a** SeqMonk screenshot of the DNA methylation profiles of the CGIs at the locus *Sfi1* in chromosome 11. Each color-coded vertical bar represents the methylation value of a differentially methylated CGI. Genes and oocyte mRNA are shown in red or blue depending on their direction of transcription (forward or reverse, respectively). **b** Box-whisker plot showing the DNA methylation levels at 28 maternally imprinted germline differentially methylated regions (gDMRs) in each replicate. In the plots, the line across the middle of the box shows the median, the upper and lower extremities of the box show the 25th and 75th percentile of the set of data, and the upper and lower black whiskers show the median plus/minus the interquartile (25–75%) range multiplied by 2. Individual points that fall outside this range are shown as filled circles and represent single outlier tiles. **c** DNA methylation levels at the Nespas-Gnasxl gDMR for each sample. **d** SeqMonk screenshot of the DNA methylation distribution (100-CpG tiles quantified) in relation to the gene structure of *Dnmt1*. The data for the replicates is combined into the tracks labeled IFC, SO, SOA, and IV. Each color-coded bar represents the methylation value of a non-overlapping 100-CpG tile. The direction of transcription is represented by the arrows. The promoter of the oocyte transcript is marked with a black bar

The specific CGI analysis revealed that there were 107 differentially methylated CGIs between SO and SOA (Additional file 14: Table S7) but few had greater than 20% methylation differences (Table 3). However, looking at the list of 110 altered CGIs, we found that SFI1 centrin binding protein gene (*Sfi1*) was the most affected gene, with ten of its 13 intragenic CGIs significantly hypomethylated, including the CGI at the promoter region (Fig. 5a). The methylation average of all CGIs in the *Sfi1* locus was 40.52% for SO and 51.71% for SOA. *Sfi1* encodes a centrosome protein required for proper mitotic spindle assembly, whose deletion results in G2/M cell-cycle arrest [55]. Importantly, it has been observed that *Sfi1* is one of the 23 loci that resist the wave of demethylation in primordial germ cells (PGCs) [56]. In addition, *Sfi1* was found to be methylated in gametes and blastocysts but also in 5-dpp (days post-partum) non-growing oocytes, oocytes lacking DNMT3A or DNMT3L and PGCs [22, 56], suggesting an incomplete demethylation during PGC reprogramming and preimplantation development. Finally, we also found differences in the unique intragenic CGI of the *Zscan10* gene (37.2% vs. 50.2%, in SO and SOA, respectively). *Zscan10*, also known as *Zfp206*, codes for a transcription factor that acts as a positive regulator of pluripotency in embryonic stem cells (ESC) and preimplantation embryos by interacting with Oct4 and Sox2 [57, 58]. Kawashima et al. (2012) [59] found that this specific CGI regulates the gene expression of the gene during mouse brain development, and that its aberrant hypomethylation was associated with human neuroblastomas, especially in patients with poor prognosis.

Because hierarchical cluster analysis showed that IFC followed the same trend as SO for these differentially methylated sites (Fig. 4b), we also interrogated the DMRs between IFC and SOA. We found that IFC had 7635 significantly differential methylated tiles (*p* < 0.05, 3.9% of total 197,565 informative tiles) with ≥ 20% methylation difference: 4158 hypomethylated and 3477 hypermentylated (Additional file 16: Table S9). Clustering of the hypomethylated tiles by position revealed that the most affected genes were *Msi2* and *Sox5* (Additional file 17: Table S10), which were also among the most affected in the SO vs. SOA and IFC vs. SO comparisons, respectively. All hypomethylation differences between IFC and SO were also present between IFC and SOA oocytes, suggesting that the differences are the result of the in vitro growth conditions (Fig. 4d). Clustering the hypermethylated tiles revealed that the most affected gene was *Tcf4*, similar to what was observed in the SO vs. SOA comparison (Additional file 17: Table S10). These results suggested that there would be genes commonly affected in the SO vs. SOA and IFC vs. SOA comparisons, which might be related to sexual maturity of oocytes. Indeed, we identified 352 genes commonly hypermethylated in SO vs. SOA and IFC vs. SOA (Fig. 4e, Additional file 18: Table S11). We performed a GO enrichment analysis and observed that these genes were enriched in regulation of nervous system development processes (Additional file 18: Table S11).

### Neither in vitro growth or hormonal stimulation alter methylation at imprinted germline differential methylated regions

Because of the importance of establishing methylation correctly at imprinted genes, we evaluated whether methylation at imprinted gDMRs was affected by in vitro culture or hormonal stimulation of prepubertal oocytes. All samples presented high percentages of methylation at the 28 maternally methylated gDMRs assessed and sample IV1 showed the greatest variation and tendency for reduced methylation (Fig. 5b). Logistic regression comparison of all groups revealed that IV had lower methylation (≥ 20% methylation difference, *p* value < 0.05) at a single gDMR, at the guanine nucleotide binding protein alpha stimulating (*Gnas*), than the other groups (Fig. 5c). Since this seemed to be driven by a single sample IV1, and our IV group was the in vivo reference for the manipulations (IFC, SO, and SOA), we did not consider the difference biologically relevant. Moreover, as noted above, IV1 was suspected to have slight somatic cell DNA contamination, which might reduce the measured gDMR methylation level.

### DNA methylation at genes for maternal effect proteins involved in imprinting maintenance

Our previous results demonstrated a reduction in methylation at the gDMRs of *H19*, *Snrpn*, and *Mest* in blastocysts generated by fertilization of IFC oocytes [34]. For this reason, we also investigated the methylation status of CGIs, promoters, and gene bodies of genes related to imprinting establishment and/or maintenance during preimplantation development. Variations in methylation of these loci could relate to transcriptional differences in oocytes, or affect the regulation of these genes in early preimplantation embryos. The genes examined included the DNA methyltransferases (*Dnmt1*; Fig. 5d, *Dnmt3a* and *Dnmt3L*); developmental pluripotency-associated protein 3 (*Dppa3*, also known as *Stella*); Tet methylcytosine dioxygenase 3 (*Tet3*); zinc finger protein 57 (*Zfp57*); the tripartite motif-containing 28 (*Trim28/Kap1*); the methyl-CpG binding domain protein 3 (*Mbd3*); the histone H3 lysine 9 methyltransferases *Setdb1*, G9a (*Ehmt2*) and G9a-like protein GLP (*Ehmt1*). All groups showed similar methylation percentages at promoters, CGIs, and gene bodies of all selected genes (Additional file 7: Figure S7).

## Discussion

Here, we provide the first whole-genome DNA methylation maps of mouse MII oocytes obtained after natural ovulation (IV) and after IFC. We also evaluated methylation in hormonally stimulated oocytes obtained from prepubertal mice (SO), results that could be of relevance for fertility preservation strategies in prepubertal girls. Foremost, we observed that global DNA methylation was similar in all groups: no significant differences were found globally at genomic annotations such as gene bodies, intergenic regions, promoters, CGIs, or repetitive elements. Similarly, methylation at the hypermethylated and hypomethylated domains characteristic of the oocyte was not significantly different at a global level. Therefore, regardless of the treatment that the oocyte is exposed to or its sexual maturity, the genomic DNA methylation pattern is strongly conserved. However, PCA did cluster the oocytes by group, indicating some degree of consistent variation by treatment. Despite relatively low sequencing depth of individual replicates, we were able to identify reproducible methylation differences by applying stringent cut-offs (≥ 20%); in addition, the finding of clustered methylation changes across whole genes provides assurance that there are genuine and biologically meaningful effects. In brief, we detected significant differences between IFC and age-matched SO oocytes, and between prepubertal and adult superovulated oocytes (SO vs SOA): the methylation differences between SOA and in vivo derived oocytes (IV) were very limited and generally dispersed through the genome. The minimal differences between SOA and IV could represent a background rate of false discovery in our pairwise comparisons, lending further support to the identification of bona fide methylation differences in the other comparisons.

We compared oocytes obtained from an established IFC system from the early preantral stage [39] with in vivo grown but superovulated age-matched oocytes (SO). This identified hypomethylation in IFC oocytes in a very low percentage of the genome (0.41%), but in regions that were also found hypomethylated when compared to SOA, suggesting that the differences are the result of the in vitro growth conditions. The affected genes were involved in nervous system development, but also in calcium channel activity, focal adhesion, and Ras and MAPK signaling. We also found some specific hypomethylated CGIs that overlapped promoter regions of important genes for embryo development such as fibroblast growth factor receptor 1 (*Fgfr1*), PHD finger protein 19 (*Phf19*), or CCR4-NOT transcription complex (*Cnot7*). Recently, it has been demonstrated that FGFR1 is crucial for trophectoderm development and blastocyst implantation [60]. PHF19 is a Polycomb-like (PCL) protein necessary to recruit polycomb repressive complex 2 (PRC2) to CpG islands and mediate transcriptional repression [61]. *Cnot* is a dormant maternal mRNAs that regulates deadenylation and degradation of maternal transcripts during oocyte maturation [62]. CGIs are interesting regions to design targeted pyrosequencing assays because the CpGs are less dispersed than the CpGs across genes. Therefore, we propose that these CGIs could be used in the future as DNA methylation markers to evaluate the improvement of the follicle culture techniques.

The oocyte gains methylation in the latter phases of growth, mostly at transcriptionally active gene bodies [23]. The final diameter of IFC oocytes at the MII stage was lower than in vivo grown mature oocytes, which might suggest that methylation acquisition in IFC oocytes is not complete by the time of the ovulation stimulus. This seems unlikely, however, as we did not see a generalized reduction in methylation, unless it is the case that the few affected genes complete methylation very late in oogenesis. Instead, the limited hypomethylation observed could be caused by suboptimal oocyte growth and/or by altered transcriptional state associated with the culture conditions. It has been previously published that the transcriptome of in vitro developed oocytes from secondary follicles differs from in vivo developed oocytes [63]; however, we did not find any correspondence between the reported transcription differences and the differentially methylated genes we observed.

Superovulation affects oocyte competence, oviduct, and uterine environments, resulting in fewer fertilized oocytes becoming live offspring [35]. However, we found that superovulation of adult females resulted in very few changes in oocyte methylation compared with natural ovulation (< 0.28% tiles with ≥ 20% difference). This extends previous findings of normal methylation acquisition at imprinted genes in superovulated mouse oocytes [64]. The essentially normal methylation pattern of SOA oocytes would be expected if all methylation is in place in the transcriptionally arrested, fully grown GV oocytes recruited by hormonal stimulation of the adult ovary; it also indicates that the effects on oocyte competence are unlikely to be related to methylation acquisition.

The greatest effect on oocyte methylation was detected in superovulation of prepubertal females, with the number of differential methylated loci between prepubertal and adult stimulated oocytes (1.05% tiles with ≥ 20% difference) higher even than between in vitro and in vivo oocyte development. In adults, the population of early antral follicles is more heterogeneous as a result of the fluctuating central hormonal control. DNA methylation alterations detected in oocytes from sexually immature females were associated with over 40 genes, mostly being hypermethylated. Again, these gene-body differences provide evidence for epigenetic differences between the first wave and later populations of fully grown oocytes, which could reflect the different hormonal environments of the immature and mature ovary. The alternative possibility that priming prepubertal oocytes with eCG causes aberrant methylation at a defined set of genes seems less likely, given our observation that hormonal priming has little or no effect on methylation in adult oocytes.

Compared to SOA, we found 377 genes with differentially methylated regions in both IFC and SO. Again, the most significant biological processes enriched in these DMR were related to nervous system development and neurogenesis. We also found hypomethylation at intragenic CGIs of *Sfi1*, one of the few loci that showed incomplete demethylation during PGC reprogramming and preimplantation development. Therefore, further experiments on embryos produced from prepubertal oocytes should also investigate whether oocyte-derived DNA methylation at non-imprinted sequences is retained during pre- and post-implantation development and whether this maternal methylation may affect normal development.

The genome-wide analysis allowed us to interrogate all known maternally and paternally methylated imprinted gDMRs. This revealed that in vitro culture, superovulation or sexual immaturity did not affect DNA methylation at imprinted loci, confirming previous studies that imprinted DNA methylation acquisition in oocytes is a robust process that is not deregulated by superovulation [61], IFC [26, 27], or sexual maturity in superovulated mice, providing reassurance for human clinical ART practice. While DNA methylation establishment at imprinted gDMRs in the oocyte remains unaltered, there is accumulating evidence that superovulation and IFC can affect maintenance of gDMR methylation during early embryo development by affecting other aspects of oocyte quality [34–37]. In our analysis, we did not detect methylation alterations at genes known to be involved in methylation establishment and maintenance, consistent with no significant effect of ART or sexual maturity on their expression. These results are consistent with previous findings of similar transcript abundance for a subset of these genes in in vivo and in vitro developed oocytes [26, 34]. Further experiments will need to evaluate whether loss of gDMR methylation in blastocysts could be related to altered protein abundance or nuclear localization of those factors. Recently, Han et al. [65] found that mouse oocytes from high-fat diet fed female mice had a reduction of the Dppa3/Stella protein required to protect the maternal genome from demethylation in the zygote, in the absence of an associated change in transcript abundance.

## Conclusions

In conclusion, our genome-wide analysis shows that IFC is associated with altered methylation at specific set of loci. DNA methylation of superovulated prepubertal oocytes differs from that of superovulated adult oocytes, whereas oocytes from superovulated adult females differ very little from naturally ovulated oocytes. Importantly, we show that regions other than imprinted gDMRs are susceptible to methylation changes associated with ART and/or sexual immaturity in mouse oocytes. Future studies need to assess whether these specific methylation changes are physiologically significant and contribute to the reduced developmental capacity of IFC oocytes, and whether new culture approaches such as 3D systems, by working toward a more appropriate niche using bioprinting, might further improve the epigenetic signature of in vitro-grown oocytes.

## Methods

### Animals

This study was performed with F1 (C57BL/6JXCBA/Ca) females, housed and bred according to European and national standards for animal care. The mice were purchased from Charles River Laboratories (Saint Germain Nuelles, France). Experimental groups, age of females, and biological replicates are illustrated in Fig. 1a.

### In vitro follicle culture

MII oocytes were obtained from early preantral follicles as described previously (Cortvrindt and Smitz 2002; Anckaert et al. 2013b). Follicles of 110–130 μm in diameter were mechanically isolated from ovaries of 48 13-day-old females in Leibovitz L15 medium (Invitrogen). Follicle culture medium consisted of α-minimal essential medium (Invitrogen) supplemented with 5% heat-inactivated fetal bovine serum, 5 μg/ml of insulin, 5 μg/ml of transferrin, 5 ng/ml of selenium (ITS; Sigma Aldrich), and 10 IU/L of recombinant follicle-stimulating hormone (r-FSH; Gonal-F®, Serono). Follicles were individually cultured until the antral stage in an incubator at 37 °C, 100% humidity, and 5% carbon dioxide in air. Part of the medium (30 μl) was refreshed at days 3, 6, and 9. At the end of the day 9, an ovulatory stimulus was given with 1.2 IU/ml of recombinant human chorionic gonadotropin (r-hCG; Ovitrelle, Serono) supplemented with 4 ng/ml of recombinant epidermal growth factor (r-EGF) (Roche Diagnostics). Approximately 18 h after r-hCG/r-EGF administration (day 10) cumulus-oocyte complexes (COCs) containing MII oocytes were available for denudation with hyaluronidase. With a fine glass pipette, oocytes were washed several times in Leibovitz L15 medium and pictures were taken in order to measure their diameter. When no more cumulus cells were evident, oocytes were washed three times more in DNA-free sterile PBS before being photographed and snap-frozen.

### Ovarian stimulation in prepubertal and adult females

A total of 18 prepubertal 23-day-old and 24 adult 10-week-old females were used to collect superovulated MII oocytes. Females were superovulated with an intraperitoneal injection of 2.5 IU (prepubertal) or 5 IU (adult) of equine chorionic gonadotropin (eCG; Folligon, Intervet) followed 48 h later by another intraperitoneal injection of the same dose of human chorionic gonadotropin (hCG; Chorulon; Intervert). Oviducts were removed and COCs were gently released from the ampulla. COCs were denuded, photographed, washed, and stored as described above for IFC.

### Oocyte collection from unstimulated females

Natural unstimulated MII oocytes were obtained from 32 adult 10-week-old females. To improve the number of females in oestrus stage, the Whitten effect previously described in mouse was used for synchronization. Three days after the females came into contact with male pheromones, oestrus positive females were separated and COCs were retrieved from the oviduct, oocytes denuded, photographed, washed, and stored as previously described.

### Oocyte diameter measurements

The diameter of oocytes was measured before storage using the image report system of the EVOS light microscope (Life technologies) and the imaging software ImageJ. Differences in oocyte diameter were determined by non-parametric Kruskall-Wallis and Dunn’s multiple comparisons tests using GraphPad Prism version 5.0. Differences were considered significant when *p* < 0.05.

### Post bisulfite adapter tagging libraries of pooled oocytes

Due to the low amount of starting material, DNA bisulfite conversion and sequencing library preparation were performed using the post-bisulphite adapter tagging (PBAT) protocol [40] including the modifications described before [41, 66]. Additional file 8: Table S1 shows the number of pooled oocytes in each biological replicate. Library quantity and quality were assessed using Bioanalyzer 2100 (High-Sensitivity DNA chips, Applied Biosystems) and KAPA Library Quantification Kit for Illumina (KAPA Biosystems). Each library was tagged with an individual identification sequence and sequenced in a HiSeq2500 or NextSeq500.

### DNA methylation analysis

PBAT library sequence reads were mapped to the mouse genome assembly GRCm38 using Bismark software (v.0.19; Babraham Institute) and DNA methylation analysis was done using the SeqMonk software package (v.1.41; Babraham Institute). Note that mapping was done onto the C57BL/6 J reference genome, but that there are sequence variants between the C57BL/6 J and CBA/Ca genomes. Methylation assignment from PBAT data in Bismark infers C to T conversions that result from bisulphite treatment as being unmethylated and retained C sites as being methylated, therefore C > T genetic variants could be erroneously called as unmethylated. However, by reference to high-quality CBA/Ca single-nucleotide polymorphisms (SNPs) from the Mouse Genomes Project (https://www.sanger.ac.uk/science/data/mouse-genomes-project), we estimate that only 0.988% of genomic CpG sites coincide with a C > T SNP in the CBA/Ca genome, and could therefore be given a false methylation assignment. We regarded this very low rate of potential false calls as being acceptable. We also note that all females from which oocytes were derived were F1[C57BL/6 J x CBA/Ca], such that all oocytes retain both C57BL/6 J and CBA/Ca alleles; therefore, there is no genetic differences between oocytes or between groups.

To perform an unbiased analysis, non-overlapping 100-CpG tiles were defined using the read position tile generator tool and selecting 1 read count per position and 100 valid positions per window, in all the 16 individual data sets. Part of the genomic features used were already implemented in SeqMonk software while others were obtained from previously published studies and converted to the correct genome coordinates, such as oocyte-specific methylated CGIs [25] and maternal imprinted gDMRs [67]. Promoters were considered − 1000 bp from any transcription start site (TSS) obtained from the mmEPDnew, the *Mus musculus* curated promoter database. For all analysis but imprinted gDMRs and CGIs, quantitation was done using the bisulphite quantitation pipeline and one minimum count to include position and five minimum observations to include feature. For imprinted gDMR and CGI analysis, quantitation was done using the bisulphite quantitation pipeline and one minimum count to include position and 20 minimum observations to include feature. We increased the threshold for observations to 20 because we were interrogating specific loci. Except for CGIs, only informative tiles (value between 0 and 100 in all 16 datasets) were included in the analyses. Differential methylated regions (DMRs) were determined with a logistic regression, with *p* < 0.05 after correction for multiple comparisons with the Benjamini–Hochberg procedure and a minimum difference of 20% in absolute methylation. Enrichment analysis was done using STRING v10.5 software with high interaction score (0.7) and the default interaction sources [68].

## Supplementary information

**Additional file 1: Figure S1.** Correlation matrix showing that all the replicates were highly correlated (100-CpG window size tiles; value between 0 and 100 in all 16 samples; *n* = 195,170).

**Additional file 2: Figure S2.** The box-whisker plots show the global DNA methylation average of probes informative in all 16 individual datasets at different genomic features: A) Intragenic regions (*n* = 20,474); B) intergenic regions (*n* = 18,245); C) promoters (*n* = 7704). In the plots the line across the middle of the box shows the median, the upper and lower extremities of the box show the 25th and 75th percentile of the set of data, and the upper and lower black whiskers show the median plus/minus the interquartile (25–75%) range multiplied by 2. Individual points which fall outside this range are shown as filled circles, and they represent single outliers tiles. D) DNA methylation levels at repetitive elements showing the mean ± SEM.

**Additional file 3: Figure S3.** Pie-charts showing the distribution of tiles overlapping gene bodies and promoters in the total informative tiles, hypomethylated tiles and hypermethylated tiles in IFC vs. SO, SO vs. SOA and SOA vs. IV comparisons. Differences were evaluated using Chi-square test and considered significant when *p* < 0.05.

**Additional file 4: Figure S4.** SeqMonk screenshots of the DNA methylation profiles of the hypomethylated loci *Myo16* (A, 13 tiles with more than 20% methylation difference) and *Elt4* (B, 14 tiles with more than 20% methylation difference) in IFC compared to SO. Each color-coded bar represents the methylation value of a non-overlapping 100-CpG tile. Genes and oocyte mRNA are shown in red or blue depending on their direction of transcription (forward or reverse, respectively). The track labeled mRNA represents the oocyte transcriptome annotaton from Veselovska et al. [24].

**Additional file 5: Figure S5.** SeqMonk screenshots of the DNA methylation profiles of the hypermethylated loci *Prdm16* (A, 7 tiles with more than 20% methylation difference) and *Soga1* (B, 6 tiles with more than 20% methylation difference) in IFC compared to SO. Each color-coded bar represents the methylation value of a non-overlapping 100-CpG tile. Genes and oocyte mRNA are shown in red or blue depending on their direction of transcription (forward or reverse, respectively). The track labeled mRNA represents the oocyte transcriptome annotaton from Veselovska et al. [24].

**Additional file 6: Figure S6.** SeqMonk screenshots of the DNA methylation profiles of the hypermethylated loci *Zfp521* (A, 26 tiles with more than 20% methylation difference) and *Atrnl1* (B, 22 tiles with more than 20% methylation difference) in SO compared to SOA. Each color-coded bar represents the methylation value of a non-overlapping 100-CpG tile. Genes and oocyte mRNA are shown in red or blue depending on their direction of transcription (forward or reverse, respectively). The track labeled mRNA represents the oocyte transcriptome annotaton from Veselovska et al. [24].

**Additional file 7: Figure S7.** SeqMonk screenshots of the DNA methylation distribution at gene structure of ten important maternal effect proteins for DNA methylation establishment and maintenance. The location of the gene and the direction of transcription it is represented with the arrows. The promoter of the oocyte transcript is marked with a black star on the TSS (transcription start site) track. The data for the replicates is combined into the tracks labeled IFC, SO, SOA and IV. Each color-coded bar represents the methylation value of a non-overlapping 100-CpG tile. The track labeled mRNA represents the oocyte transcriptome annotaton from Veselovska et al. [24].

**Additional file 8: Table S1.** Summary of all PBAT libraries generated for this study, including the following information: the approximate number oocytes used for each library, number of uniquely mappable reads, mapping efficiency, duplication rate and methylation percentage at CpG, CHG and CHH regions.

**Additional file 9: Table S2.** Sequencing statistics for each experimental group including the number of CpGs covered by more than 1, 3 or 5 reads.

**Additional file 10: Table S3.** Differentially hypomethylated tiles (*n*=829) and hypermethylated tiles (*n*=702) with more than 20% methylation difference in IFC compared to SO. Each tile contains information about the genome location, overlapping gene, Ensembl ID, gene description and methylation percentage in each sample. Differentially methylated CGIs between IFC and SO (*n*=85).

**Additional file 11: Table S4.** Specific loci in IFC compared to SO that contained multiple differential hypo- or hypermethylated tiles (> 20% methylation difference). Enriched biological processes of hypomethylated genes in IFC compared to SO (*n* = 17), including the proteins involved in each GO term.

**Additional file 12: Table S5.** Differentially hypomethylated tiles (*n*=110) and hypermethylated tiles (*n*=455) with more than 20% methylation difference in SOA compared to IV. Each tile contains information about the genome location, overlapping gene, Ensembl ID, gene description and methylation percentage in each sample. Differentially methylated CGIs between SOA and IV (*n*=60).

**Additional file 13: Table S6.** Specific loci in SOA compared to IV that contained multiple differential hypo- or hypermethylated tiles (> 20% methylation difference). Enriched biological processes of hypermethylated promoters in SOA vs IV (20% difference of methylation, *n*=128).

**Additional file 14: Table S7.** Differentially hypomethylated tiles (*n*=48) and hypermethylated tiles (*n*=2031) with more than 20% methylation difference in SO compared to SOA. Each tile contains information about the genome location, overlapping gene, Ensembl ID, gene description and methylation percentage in each sample. Differentially methylated CGIs between SO and SOA (*n*=107)

**Additional file 15: Table S8.** Specific loci in SO vs. SOA comparison that contained multiple differential hypo- or hypermethylated tiles (more than 20% methylation difference). Enriched biological processes of hypermethylated genes in SO compared to SOA (*n*=40), including the proteins involved in each GO term.

**Additional file 16: Table S9.** Differentially hypomethylated tiles (*n*=4158) and hypermethylated tiles (*n*=3477) with more than 20% methylation difference in IFC compared to SOA. Each tile contains information about the genome location, overlapping gene, Ensembl ID, gene description and methylation percentage in each sample.

**Additional file 17: Table S10.** Specific loci in IFC vs. SOA comparison that contained multiple differential hypo- or hypermethylated tiles (more than 20% methylation difference).

**Additional file 18: Table S11.** Genes commonly hypermethylated or hypomethylated in all pairwise comparisons. Enriched biological processes of genes commonly affected in the SO vs. SOA and IFC vs. SOA comparisons related to sexual maturity of oocytes (*n*=352).

## Data Availability

Mapped sequence data from Bismark software have been deposited in the Gene Expression Omnibus database (GEO) under accession code GSE128656.

## Abbreviations

ARTs: Assisted reproductive technologies
AS: Angelman syndrome
BWS: Beckwith-Wiedemann syndrome
CGIs: CpG islands
COCs: Cumulus-oocyte complexes
DMRs: Differential methylated regions
eCG: Equine chorionic gonadotropin
ESC: Embryonic stem cells
gDMRs: Germline differential methylated regions
GO: Gene ontology
GV: Germinal vesicle stage
hCG: Human chorionic gonadotropin
HyperD: Hypermethylated domain
HypoD: Hypomethylated domain
IFC: In vitro follicle culture
IVM: In vitro maturation
MII: Metaphase-II stage
PB: Polar body extrusion
PBAT: Post-bisulphite adapter tagging
PC1: First principal component
PCA: Principal component analysis
PGCs: Primordial germ cells
PWS: Prader-Willi syndrome
r-EGF: Recombinant epidermal growth factor
r-hCG: Recombinant human chorionic gonadotropin
SEM: Standard error of the mean
SNPs: Single-nucleotide polymorphism
SRS: Silver-Russell syndrome
TSS: Transcription start site
