# Correction to: Genome-wide assessment of DNA methylation in mouse oocytes reveals effects associated with in vitro growth, superovulation, and sexual maturity

**DOI:** 10.1186/s13148-020-0812-0

**Published:** 2020-01-27

**Authors:** Maria Desemparats Saenz-de-Juano, Elena Ivanova, Katy Billooye, Anamaria-Cristina Herta, Johan Smitz, Gavin Kelsey, Ellen Anckaert

**Affiliations:** 1 Follicle Biology Laboratory (FOBI), UZ Brussel, Vrije Universiteit Brussel, Laarbeeklaan, Brussels, Belgium; 2 Present Address: Animal Physiology, Institute of Agricultural Sciences, ETH Zurich, Zurich, Switzerland; 3 EpigeneticsProgramme, Babraham Institute, Cambridge, CB22 3AT UK; 4 Centre for Trophoblast Research, University of Cambridge, Cambridge, CB2 3EG UK

**Correction to: Clin Epigenet**

**https://doi.org/10.1186/s13148-019-0794-y**

After publication of the original article [1], we were notified that: 

the software used to create the figures has exported them wrong so they display incomplete. Below you can find the correct version of the figures:

**Fig. 1:**
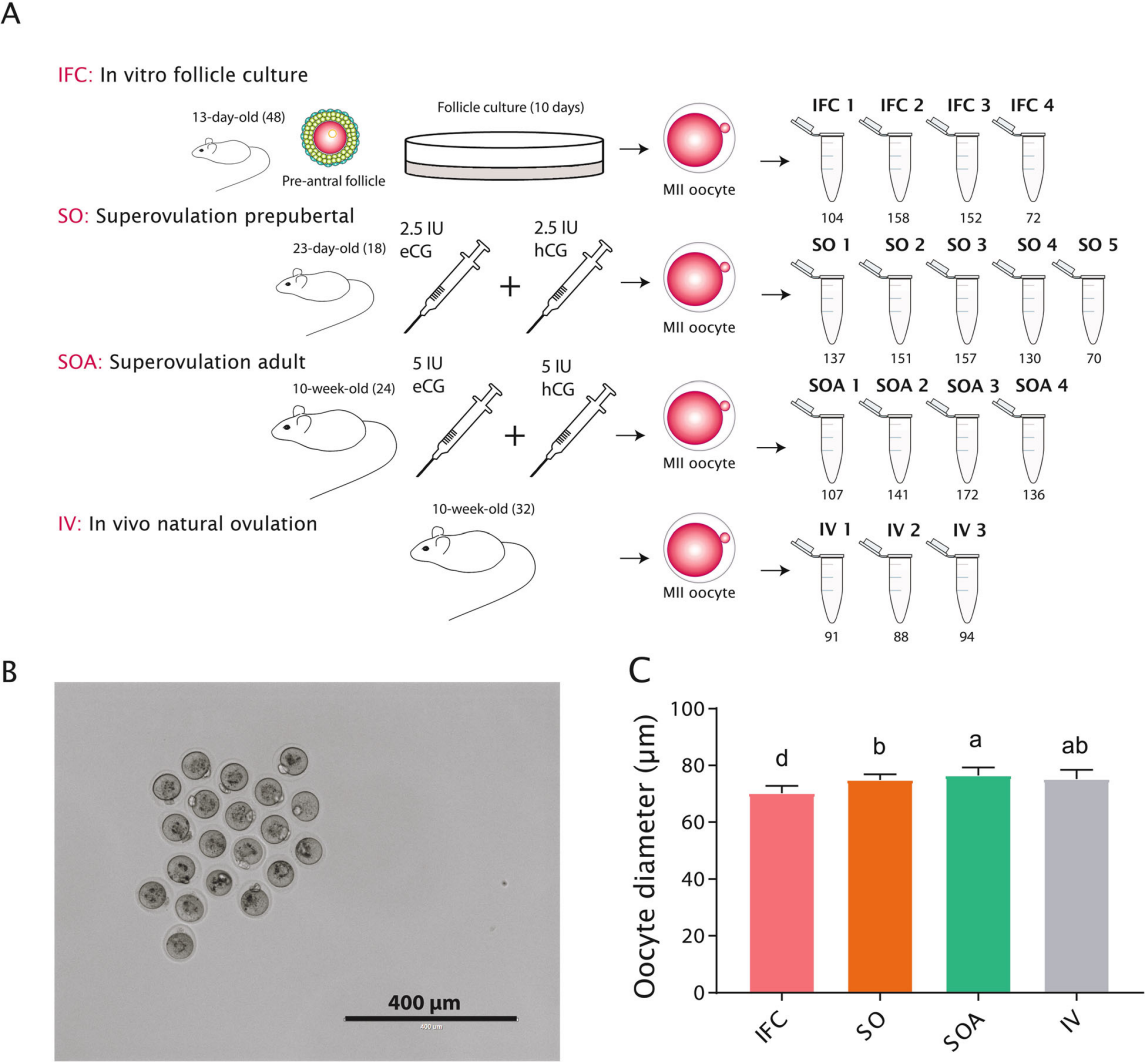
**a** Experimental groups used for the genome-wide DNA methylation analysis. The number of females used per group is indicated in brackets next to their age. The tubes represent the number of biological replicates and the number under each tube indicates the number of oocytes pooled in each sample. *MII* metaphase II, *eCG* equine chorionic gonadotropin, *hCG* human chorionic gonadotropin. **b** MII oocytes from IFC obtained after 10 days of culture. Pictures were taken before snap freezing to measure oocyte diameters. **c** Oocyte diameter per group. Bar charts show the mean and the standard deviation (SD). Lower-case letters denote significant differences (*p* < 0.05) after applying non-parametric Krustall-Wallis and Dunn’s multiple comparisons tests

**Fig. 2:**
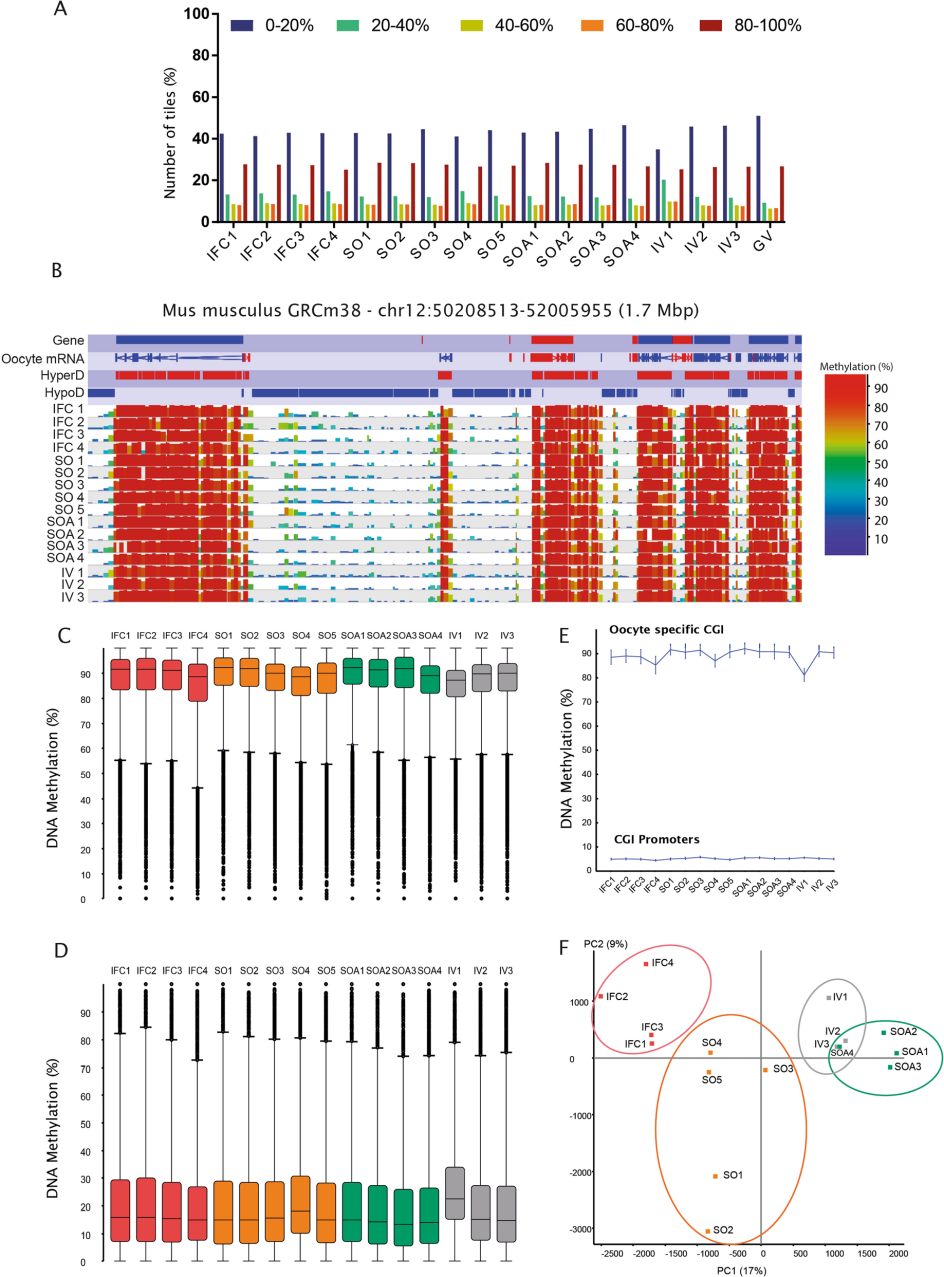
**a** Distribution of DNA methylation across the genome in 100-CpG windows in all samples compared to Germinal Vesicle (GV) oocytes from Shirane et al. [38]. **b** SeqMonk screenshot of a 1.7 Mb region of chromosome 11 depicting the hypermethylated (HyperD) and hypomethylated (HypoD) domains characteristic of the oocyte methylome in each of the 16 individual methylation datasets. Genes and oocyte mRNA are shown in red or blue depending on their direction of transcription (forward and reverse, respectively). Each color-coded vertical bar in the screenshot represents the methylation value of a non-overlapping 100 CpG tile. HypoD, HyperD, and oocyte mRNA annotation tracks are derived from Veselovska et al. [24]. **c** DNA methylation percentages at HyperD in all samples (*n* = 26,570). In the box:whiskers plot, the line across the middle of the box shows the median, the upper and lower extremities of the box show the 25th and 75th percentile of the set of data, and the upper and lower black whiskers show the median plus/minus the interquartile (25–75%) range multiplied by 2. Individual points which fall outside this range are shown as filled circles, and represent single outlier tiles. **d** Box:whisker plot showing the DNA methylation percentages at HypoD (*n* = 38,739). **e** DNA methylation percentages of CpG Islands (CGI) located at promoters (*n* = 11,542) and CGIs highly methylated in oocytes (*n* = 2014). Each point represents the mean value along with error bars indicating the 95% confidence interval for the measure. **f** Principal component analysis (PCA) of informative 100-CpG tiles (value between 0 and 100 in all 16 samples; *n* = 195,170) shows how biological replicates cluster together within each group and differently between conditions

**Fig. 3:**
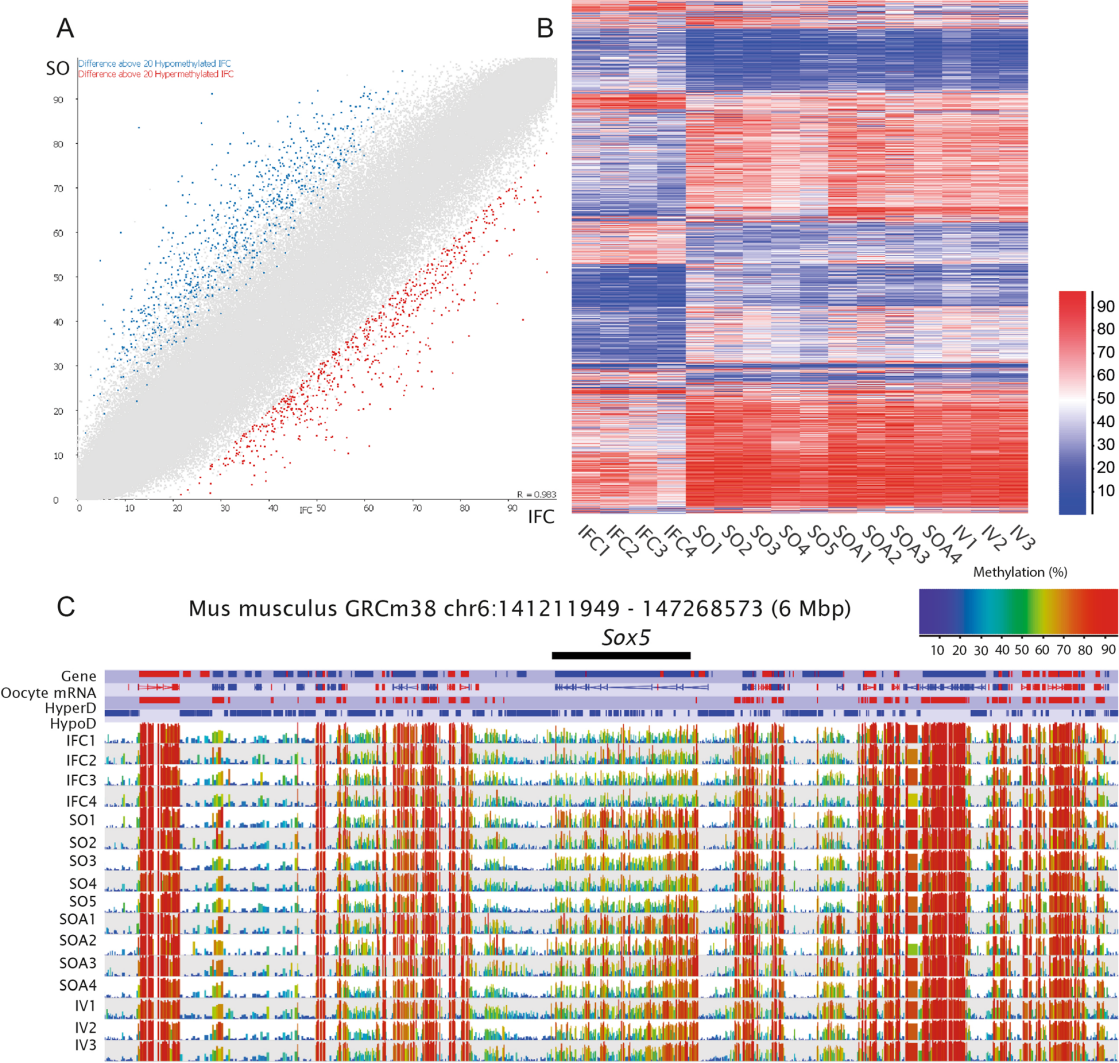
**a** Scatterplot for informative tiles (100 CpG window size, *n* = 195,170) in both IFC and SO. Data from replicates are pooled. Differentially methylated tiles (*p* < 0.05) identified by logistic regression and with a methylation difference of ≥ 20% are highlighted in blue or red (hypomethylated in IFC and hypermethylated in IFC, respectively). **b** Heat map after unsupervised hierarchical clustering of all differentially methylated tiles (*p* < 0.05, 100-CpG window size, *n* = 6362) between IFC and SO. The heatmap shows how biological replicates were consistent within groups and IFC differed in a similar way from SO, SOA, and IV for these differentially methylated sites. **c** SeqMonk screenshot of a 6 Mbp region of chromosome 6 showing methylation at the *Sox5* locus, with 18 hypomethylated tiles in IFC. Each color-coded vertical bar in the screenshot represents the methylation value of a non-overlapping 100-CpG tile. Genes and oocyte mRNA are shown in red or blue depending on their direction of transcription (forward or reverse, respectively)

**Fig. 4:**
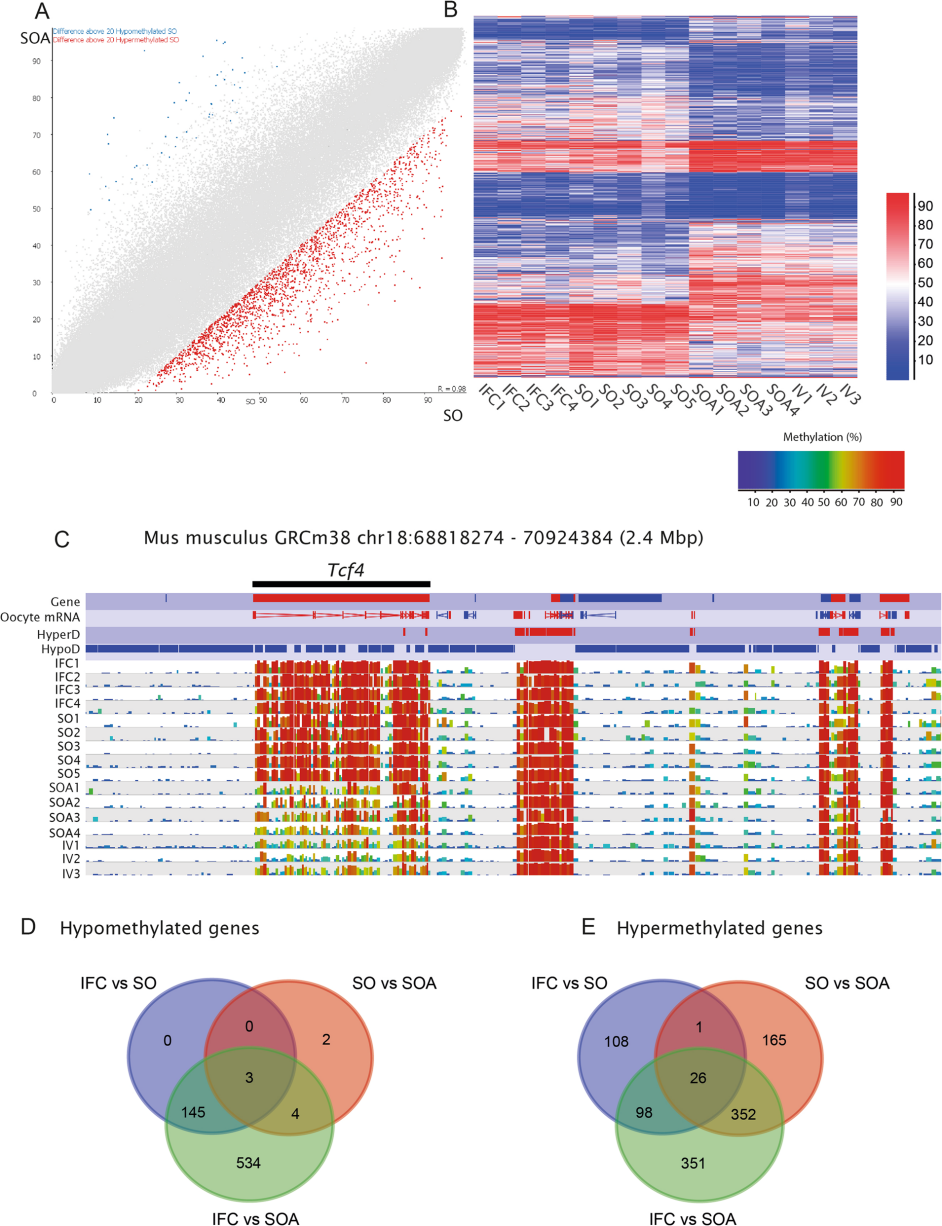
**a** Scatterplot for common informative tiles (100 CpG window size, *n* = 195,170 between SO and SOA. Data from replicates are pooled. Differentially methylated tiles (*p* < 0.05) identified by logistic regression and with a methylation difference of ≥ 20% are highlighted in blue or red (hypomethylated in IFC and hypermethylated in IFC, respectively). **b** Heat map after unsupervised hierarchical clustering of all differentially methylated tiles (*p* < 0.05, 100 CpG window size, *n* = 14,795 between SO and SOA. The heatmap shows that the IFC group followed the same trend as SO, while the IV group was similar to SOA for these differentially methylated sites. **c** SeqMonk screenshot showing the methylation levels at Tcf4 locus (with 28 hypermethylated tiles). Each color-coded vertical bar represents the methylation value of a non-overlapping 100-CpG tile. Genes and oocyte mRNA are shown in red or blue depending on their direction of transcription (forward or reverse, respectively). **d**, **e** Venn diagrams showing the common hypomethylated and hypermethylated genes that were affected in IFC vs. SO, SO vs. SOA, and IFC vs. SOA

**Fig. 5:**
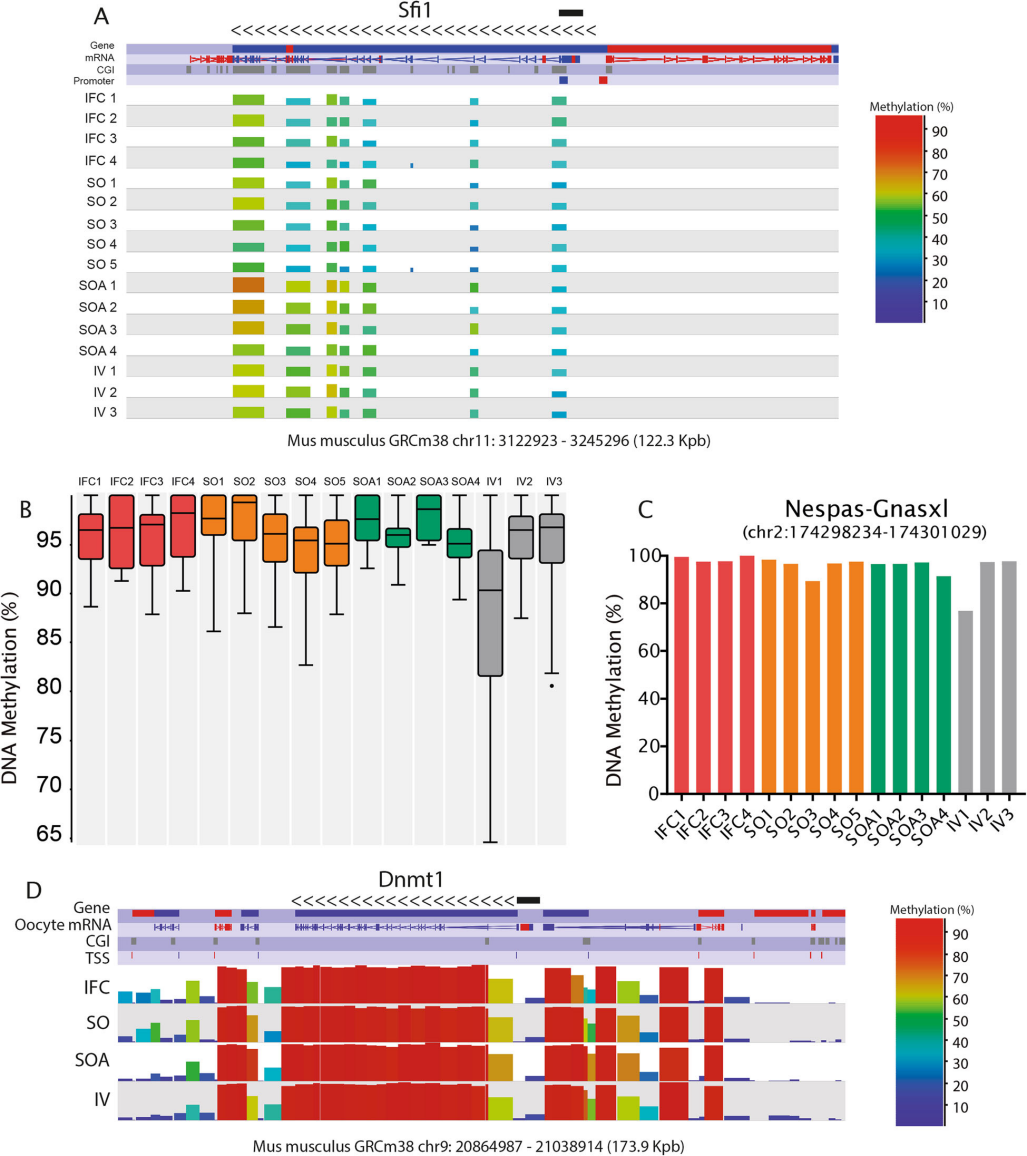
**a** SeqMonk screenshot of the DNA methylation profiles of the CGIs at the locus *Sfi1* in chromosome 11. Each color-coded vertical bar represents the methylation value of a differentially methylated CGI. Genes and oocyte mRNA are shown in red or blue depending on their direction of transcription (forward or reverse, respectively). **b** Box-whisker plot showing the DNA methylation levels at 28 maternally imprinted germline differentially methylated regions (gDMRs) in each replicate. In the plots, the line across the middle of the box shows the median, the upper and lower extremities of the box show the 25th and 75th percentile of the set of data, and the upper and lower black whiskers show the median plus/minus the interquartile (25–75%) range multiplied by 2. Individual points that fall outside this range are shown as filled circles and represent single outlier tiles. **c** DNA methylation levels at the Nespas-Gnasxl gDMR for each sample. **d** SeqMonk screenshot of the DNA methylation distribution (100-CpG tiles quantified) in relation to the gene structure of *Dnmt1*. The data for the replicates is combined into the tracks labeled IFC, SO, SOA, and IV. Each color-coded bar represents the methylation value of a non-overlapping 100-CpG tile. The direction of transcription is represented by the arrows. The promoter of the oocyte transcript is marked with a black bar

The original article has been corrected.
