# Supplementary figures and images for: Genome-wide assessment of DNA methylation in mouse oocytes reveals effects associated with in vitro growth, superovulation, and sexual maturity
Source: Clin Epigenetics. 2019 Dec 19;11:197. doi: 10.1186/s13148-019-0794-y (PMC6923880; doi:10.1186/s13148-019-0794-y)

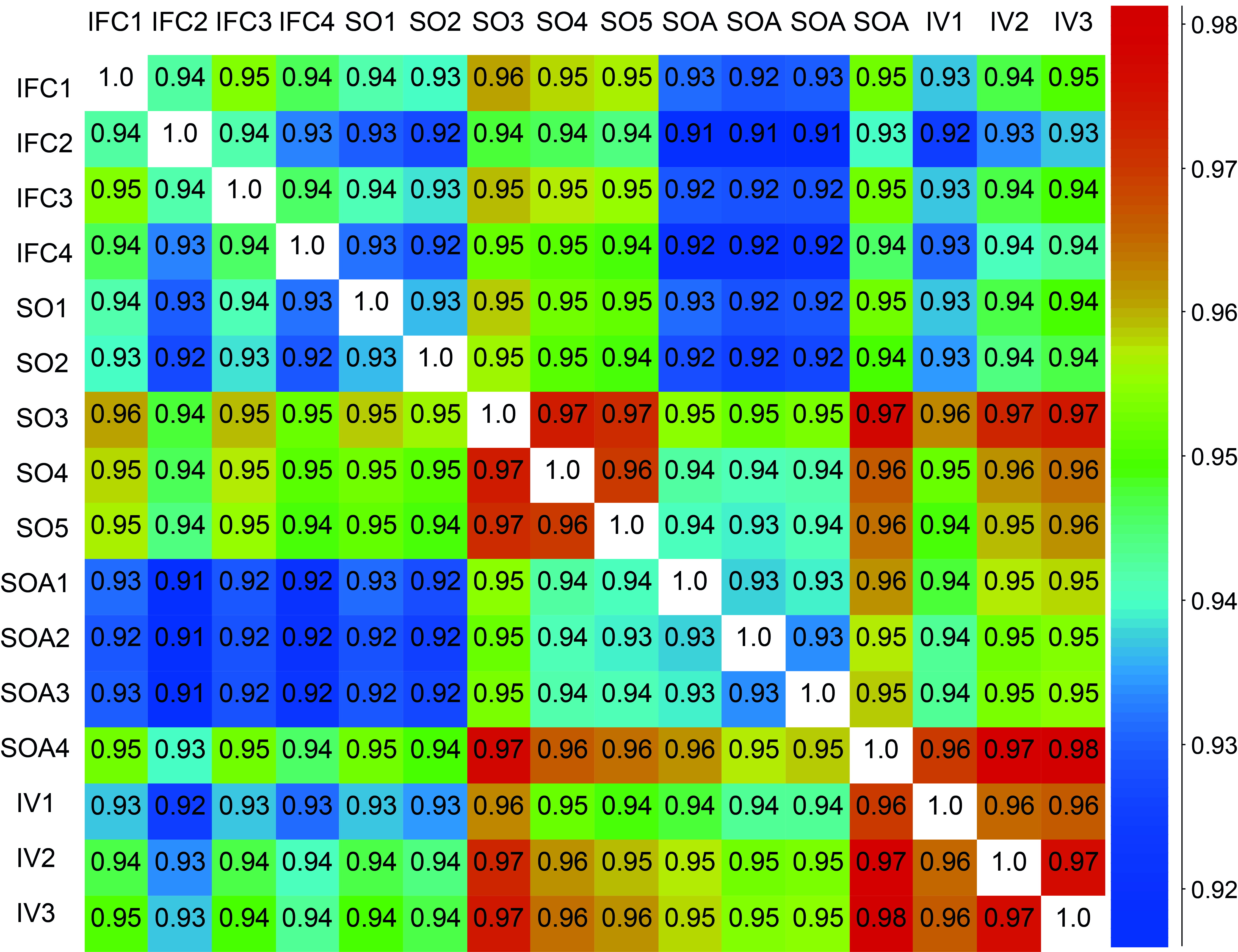

Supplement: Supplementary file 1 — Additional file 1: Figure S1. Correlation matrix showing that all the replicates were highly correlated (100-CpG window size tiles; value between 0 and 100 in all 16 samples; n = 195,170). [file 13148_2019_794_MOESM1_ESM.jpg]

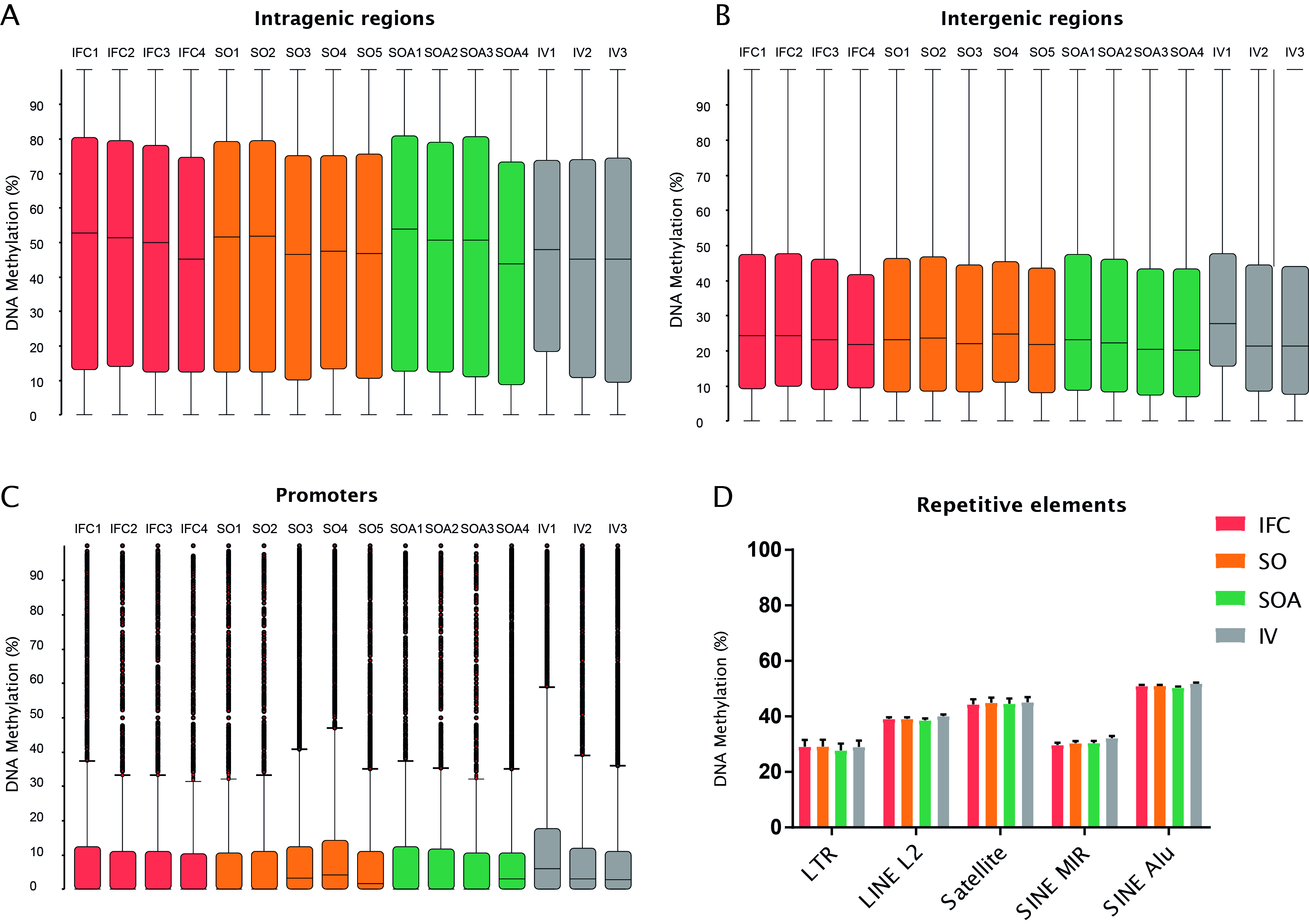

Supplement: Supplementary file 2 — Additional file 2: Figure S2. The box-whisker plots show the global DNA methylation average of probes informative in all 16 individual datasets at different genomic features: A) Intragenic regions (n = 20,474); B) intergenic regions (n = 18,245); C) promoters (n = 7704). In the plots the line across the middle of the box shows the median, the upper and lower extremities of the box show the 25th and 75th percentile of the set of data, and the upper and lower black whiskers show the median plus/minus the interquartile (25–75%) range multiplied by 2. Individual points which fall outside this range are shown as filled circles, and they represent single outliers tiles. D) DNA methylation levels at repetitive elements showing the mean ± SEM. [file 13148_2019_794_MOESM2_ESM.jpg]

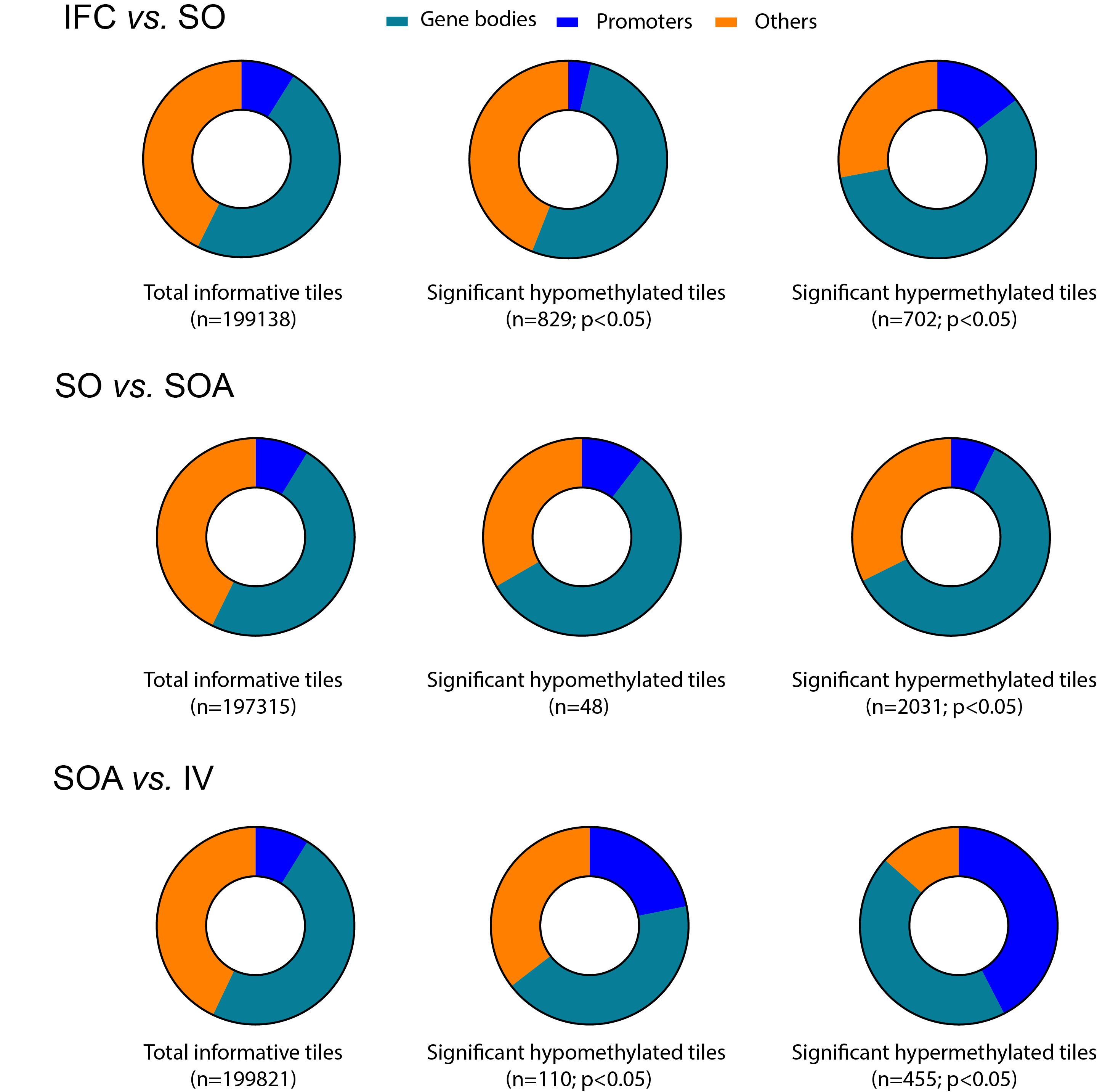

Supplement: Supplementary file 3 — Additional file 3: Figure S3. Pie-charts showing the distribution of tiles overlapping gene bodies and promoters in the total informative tiles, hypomethylated tiles and hypermethylated tiles in IFC vs. SO, SO vs. SOA and SOA vs. IV comparisons. Differences were evaluated using Chi-square test and considered significant when p < 0.05. [file 13148_2019_794_MOESM3_ESM.jpg]

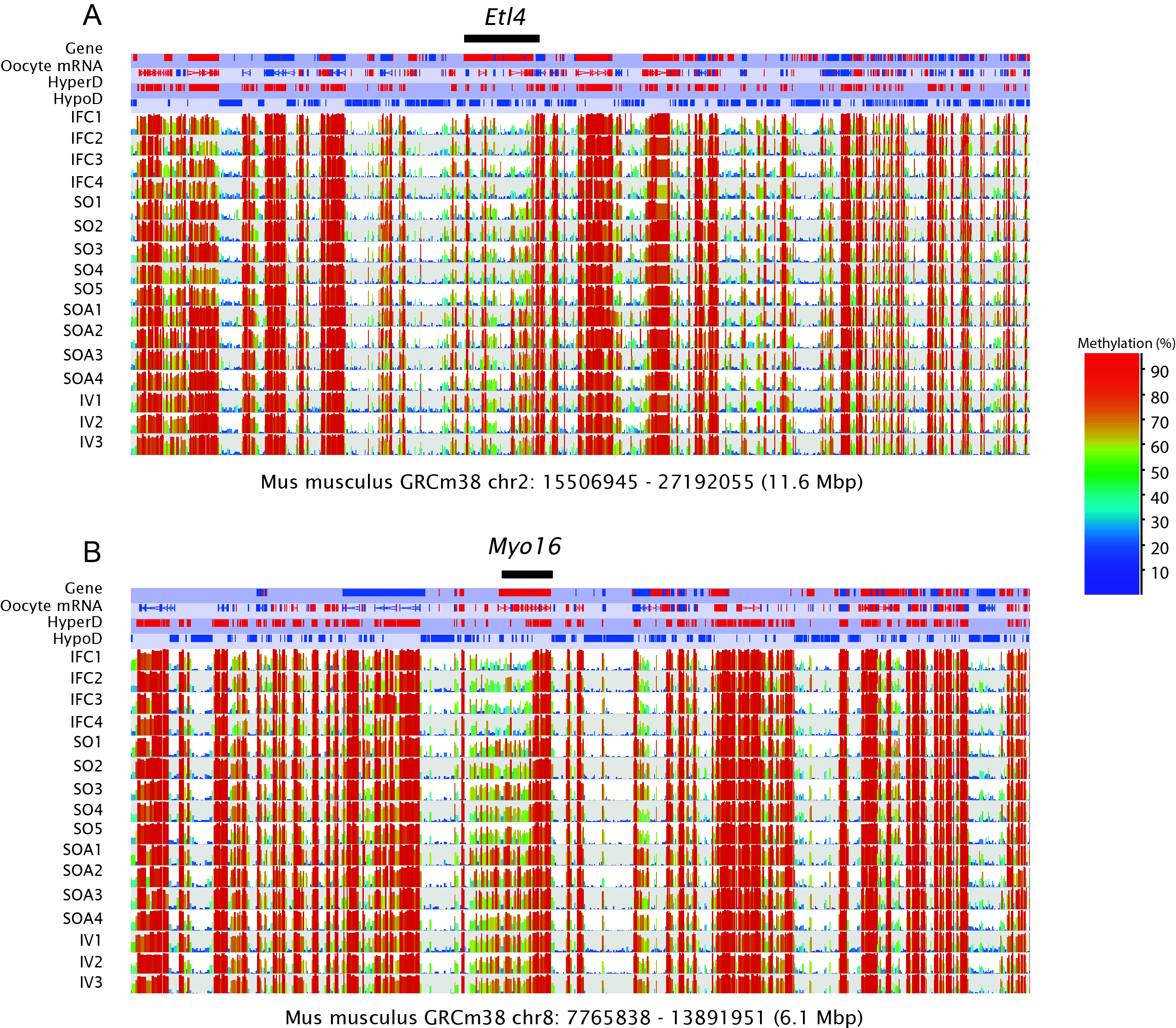

Supplement: Supplementary file 4 — Additional file 4: Figure S4. SeqMonk screenshots of the DNA methylation profiles of the hypomethylated loci Myo16 (A, 13 tiles with more than 20% methylation difference) and Elt4 (B, 14 tiles with more than 20% methylation difference) in IFC compared to SO. Each color-coded bar represents the methylation value of a non-overlapping 100-CpG tile. Genes and oocyte mRNA are shown in red or blue depending on their direction of transcription (forward or reverse, respectively). The track labeled mRNA represents the oocyte transcriptome annotaton from Veselovska et al. [24]. [file 13148_2019_794_MOESM4_ESM.jpg]

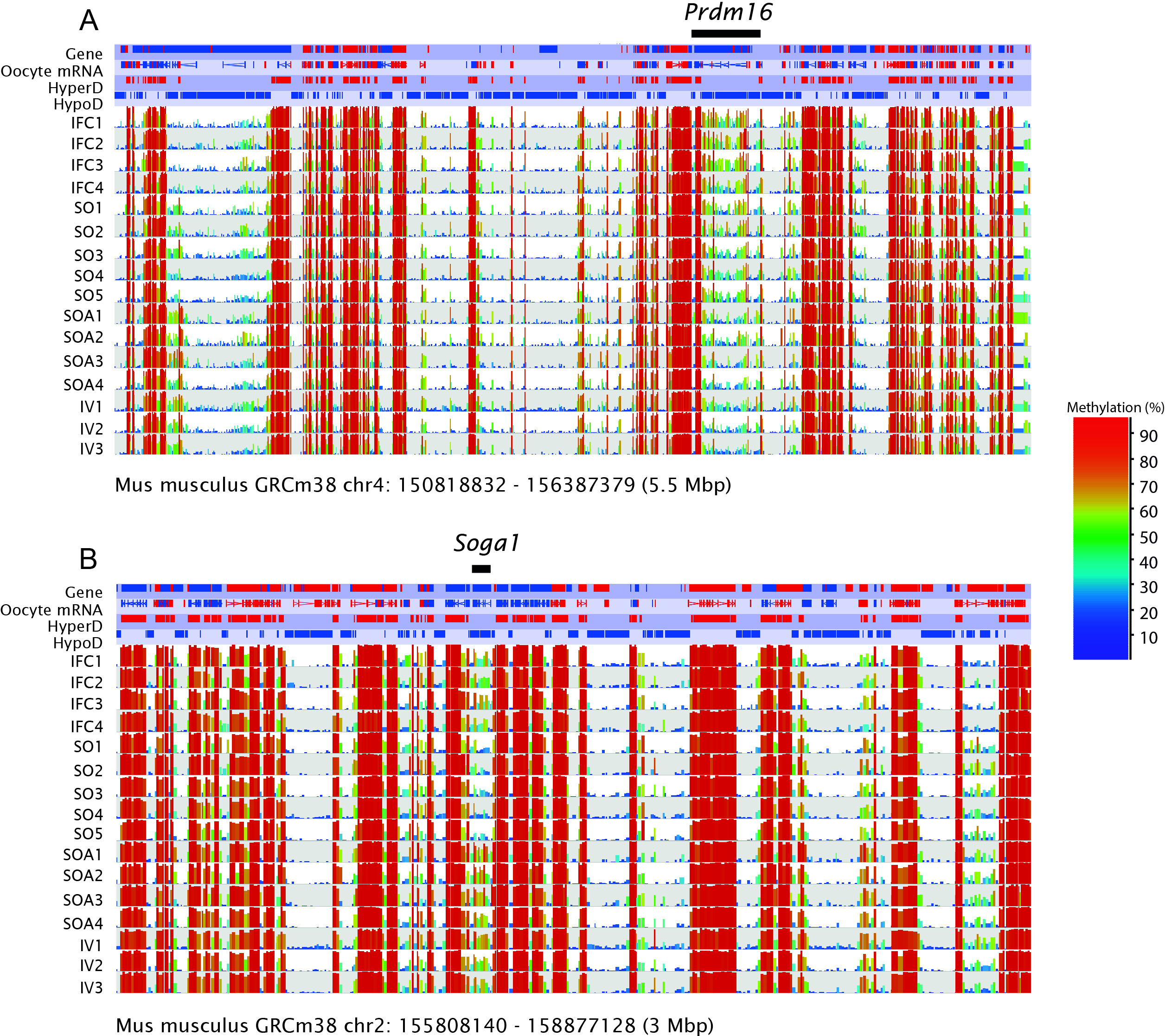

Supplement: Supplementary file 5 — Additional file 5: Figure S5. SeqMonk screenshots of the DNA methylation profiles of the hypermethylated loci Prdm16 (A, 7 tiles with more than 20% methylation difference) and Soga1 (B, 6 tiles with more than 20% methylation difference) in IFC compared to SO. Each color-coded bar represents the methylation value of a non-overlapping 100-CpG tile. Genes and oocyte mRNA are shown in red or blue depending on their direction of transcription (forward or reverse, respectively). The track labeled mRNA represents the oocyte transcriptome annotaton from Veselovska et al. [24]. [file 13148_2019_794_MOESM5_ESM.jpg]

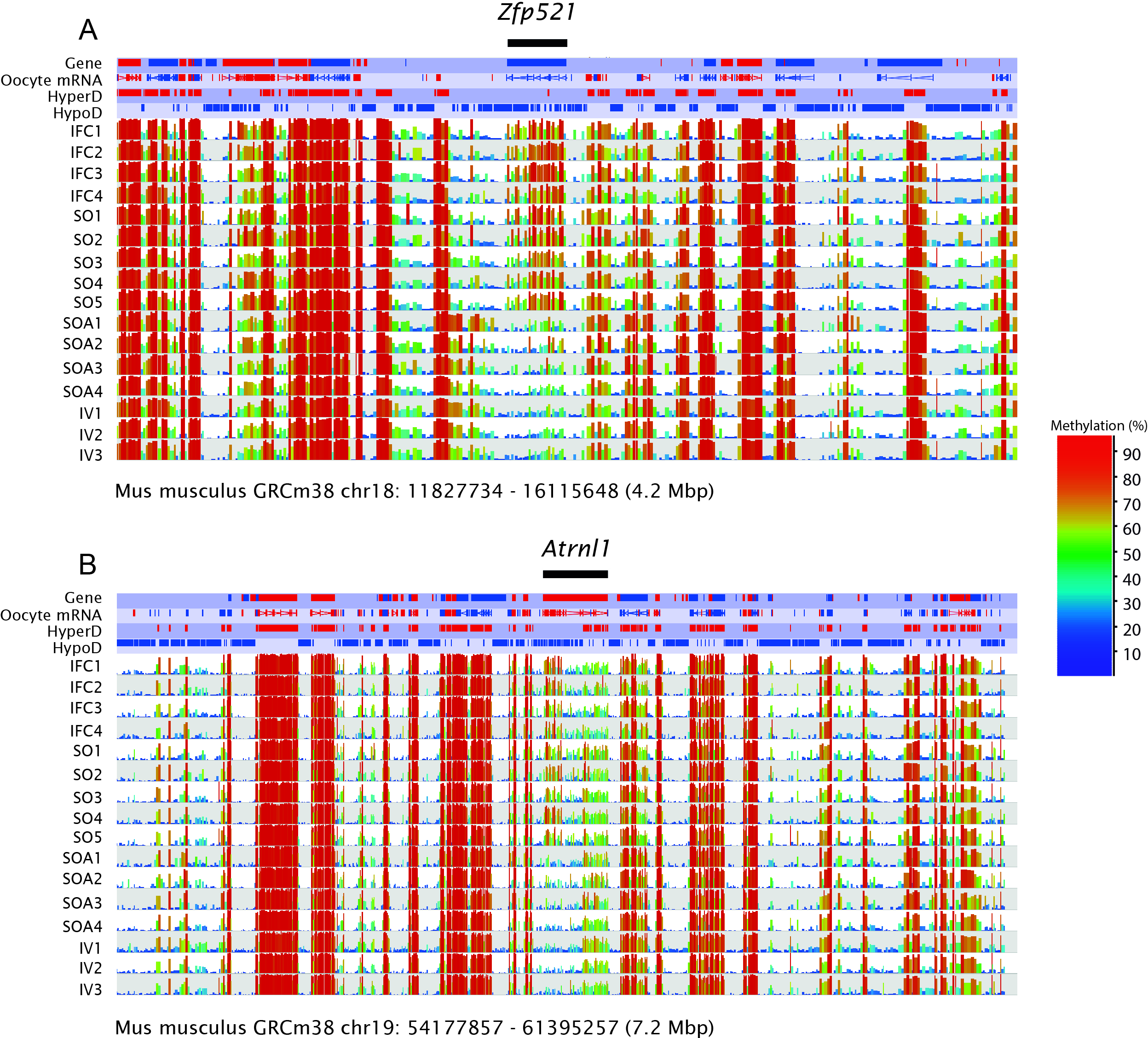

Supplement: Supplementary file 6 — Additional file 6: Figure S6. SeqMonk screenshots of the DNA methylation profiles of the hypermethylated loci Zfp521 (A, 26 tiles with more than 20% methylation difference) and Atrnl1 (B, 22 tiles with more than 20% methylation difference) in SO compared to SOA. Each color-coded bar represents the methylation value of a non-overlapping 100-CpG tile. Genes and oocyte mRNA are shown in red or blue depending on their direction of transcription (forward or reverse, respectively). The track labeled mRNA represents the oocyte transcriptome annotaton from Veselovska et al. [24]. [file 13148_2019_794_MOESM6_ESM.jpg]

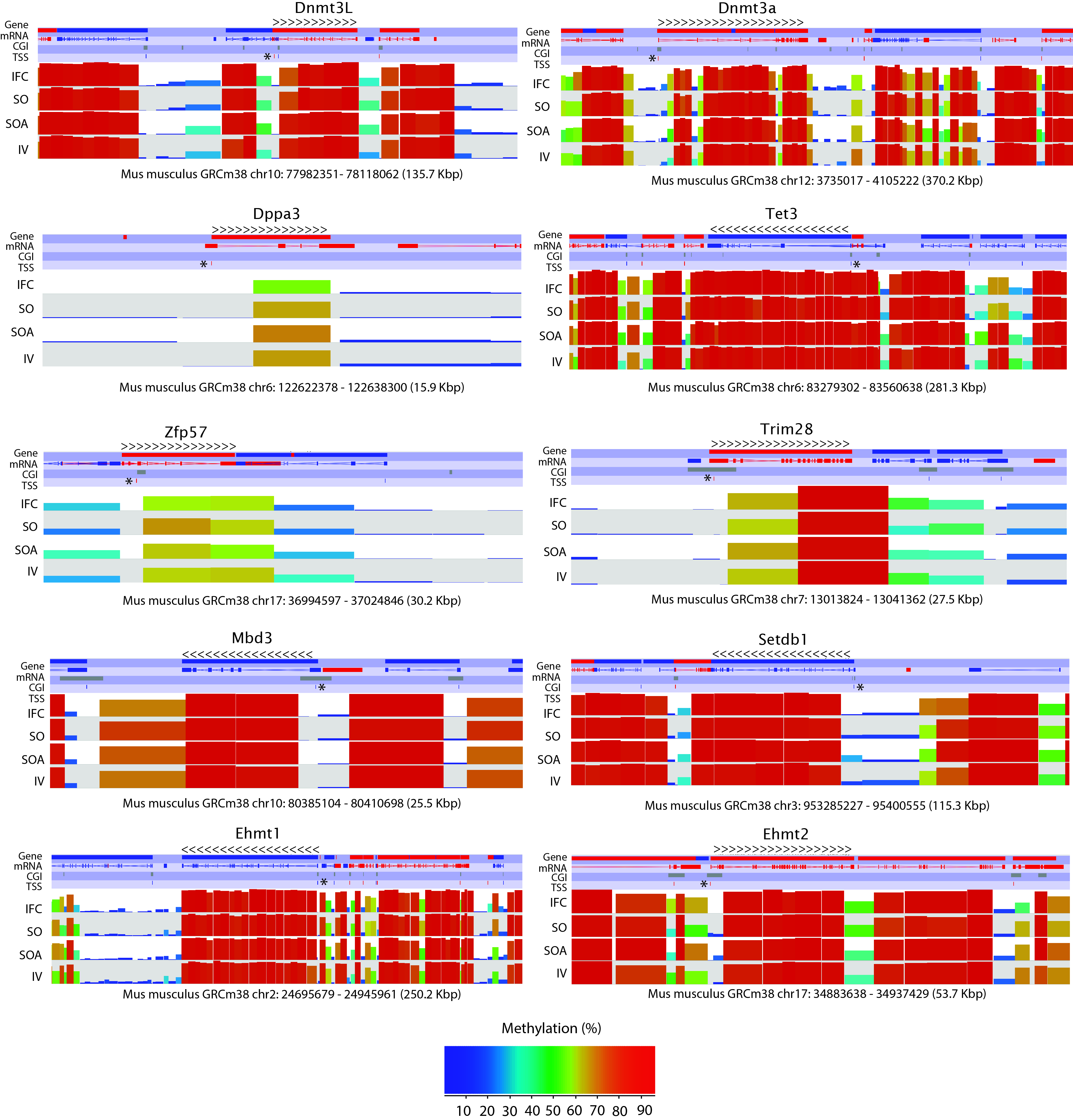

Supplement: Supplementary file 7 — Additional file 7: Figure S7. SeqMonk screenshots of the DNA methylation distribution at gene structure of ten important maternal effect proteins for DNA methylation establishment and maintenance. The location of the gene and the direction of transcription it is represented with the arrows. The promoter of the oocyte transcript is marked with a black star on the TSS (transcription start site) track. The data for the replicates is combined into the tracks labeled IFC, SO, SOA and IV. Each color-coded bar represents the methylation value of a non-overlapping 100-CpG tile. The track labeled mRNA represents the oocyte transcriptome annotaton from Veselovska et al. [24]. [file 13148_2019_794_MOESM7_ESM.jpg]
