# Supplementary material for: Genome-wide assessment of DNA methylation in mouse oocytes reveals effects associated with in vitro growth, superovulation, and sexual maturity
Source: Clin Epigenetics. 2019 Dec 19;11:197. doi: 10.1186/s13148-019-0794-y (PMC6923880; doi:10.1186/s13148-019-0794-y)
Supplement: Supplementary file 8 — Additional file 8: Table S1. Summary of all PBAT libraries generated for this study, including the following information: the approximate number oocytes used for each library, number of uniquely mappable reads, mapping efficiency, duplication rate and methylation percentage at CpG, CHG and CHH regions. [file 13148_2019_794_MOESM8_ESM.docx]

**Additional file 8: Table S1.**

Summary of all PBAT libraries generated for this study, including the following information: the approximate number oocytes used for each library, number of uniquely mappable reads, mapping efficiency, duplication rate and methylation percentage at CpG, CHG and CHH regions.

| **Library ID** | **Oocytes** | **Sequences uniquely mapped** | **Mapping efficiency, %** | **Duplication rate, %** | **CpG met %** | **CHG met %** | **CHH met %** |
| --- | --- | --- | --- | --- | --- | --- | --- |
| **IFC1** | 104 | 10,611,338 | 78.4 % | 21.6 % | 42.3% | 4.8% | 4.0% |
| **IFC2** | 158 | 6,620,518 | 86.7 % | 13. %3 | 41.8% | 4.8% | 4.1% |
| **IFC3** | 152 | 9,582,839 | 85.9 % | 14.1 % | 41.1% | 4.6% | 3.9% |
| **IFC4** | 72 | 9,430,087 | 69.5 % | 30.5 % | 37.7% | 3.7% | 3.2% |
| **SO1** | 137 | 6,809,090 | 80.7 % | 19.3 % | 41.6% | 4.9% | 4.2% |
| **SO2** | 151 | 6,490,280 | 83.3 % | 16.7 % | 41.7% | 5.0% | 4.3% |
| **SO3** | 137 | 25,694,663 | 71.0 % | 29.0 % | 39.2% | 4.6% | 3.9% |
| **SO4** | 130 | 21,255,031 | 66.7 % | 33.3 % | 40.7% | 4.6% | 3.9% |
| **SO5** | 70 | 15,494,379 | 62.1 % | 37.9 % | 38.5% | 4.4% | 3.8% |
| **SOA1** | 107 | 7,908,706 | 71.3 % | 28.7 % | 42.5% | 5.0% | 4.3% |
| **SOA2** | 141 | 6,357,771 | 80.4 % | 19.6 % | 41.3% | 5.2% | 4.5% |
| **SOA3** | 172 | 7,293,916 | 70.9 % | 29.1 % | 38.4% | 3.9% | 3.3% |
| **SOA4** | 136 | 29,532,884 | 69.1 % | 30.9 % | 38.3% | 4.5% | 3.9% |
| **IV1** | 91 | 20,452,772 | 64.2 % | 35.8 % | 42.9% | 4.2% | 3.6% |
| **IV2** | 88 | 19,890,191 | 66.9 % | 33.1 % | 38.8% | 4.4% | 3.8% |
| **IV3** | 94 | 20,920,457 | 47.0% | 53.0% | 38.8% | 4.7% | 3.9% |
