# Supplementary material for: Genome-wide assessment of DNA methylation in mouse oocytes reveals effects associated with in vitro growth, superovulation, and sexual maturity
Source: Clin Epigenetics. 2019 Dec 19;11:197. doi: 10.1186/s13148-019-0794-y (PMC6923880; doi:10.1186/s13148-019-0794-y)
Supplement: Supplementary file 9 — Additional file 9: Table S2. Sequencing statistics for each experimental group including the number of CpGs covered by more than 1, 3 or 5 reads. [file 13148_2019_794_MOESM9_ESM.docx]

**Additional file 9: Table S2.**

Sequencing statistics for each experimental group including the number of CpGs covered by more than 1, 3 or 5 reads.

|  | **> 1 read** | **Percentage of coverage** | **> 3 reads** | **Percentage of coverage** | **> 5 reads** | **Percentage of coverage** |
| --- | --- | --- | --- | --- | --- | --- |
| **IV** | 16002528 | 73.18% | 7975439 | 36.47% | 3122084 | 14.27% |
| **IFC** | 13766356 | 62.95% | 3767348 | 17.23% | 636740 | 2.91% |
| **SO** | 16925228 | 77.40% | 9764236 | 44.65% | 4483329 | 20.50% |
| **SOA** | 15369525 | 70.28% | 6348306 | 29.03% | 1794253 | 8.20% |
